# Coniferaldehyde attenuates Alzheimer's pathology *via* activation of Nrf2 and its targets

**DOI:** 10.7150/thno.36722

**Published:** 2020-01-01

**Authors:** Yaqiong Dong, Tessandra Stewart, Lidan Bai, Xue Li, Ting Xu, Jeffrey Iliff, Min Shi, Danfeng Zheng, Lan Yuan, Taotao Wei, Xiaoda Yang, Jing Zhang

**Affiliations:** 1 The State Key Laboratories of Natural and Biomimetic Drugs and Department of Chemical Biology, School of Pharmaceutical Sciences, Peking University Health Science Center, Beijing 100191, China; 2 Department of Pathology, University of Washington School of Medicine, Seattle, WA 98104, United States; 3 Department of Anesthesiology and Perioperative Medicine, Knight Cardiovascular Institute, Oregon Health & Science University, Portland, OR, United States; 4 Department of Pathology, School of Basic Medical Sciences, Peking University Health Science Center, Beijing 100191, China; 5 National Laboratory of Biomacromolecules, Institute of Biophysics, Chinese Academy of Sciences, Beijing 100101, China

**Keywords:** Alzheimer's disease, Aβ clearance, coniferaldehyde, neuroprotection, Nrf2

## Abstract

**Background**: Alzheimer's disease (AD) currently lacks a cure. Because substantial neuronal damage usually occurs before AD is advanced enough for diagnosis, the best hope for disease-modifying AD therapies likely relies on early intervention or even prevention, and targeting multiple pathways implicated in early AD pathogenesis rather than focusing exclusively on excessive production of β-amyloid (Aβ) species.

**Methods**: Coniferaldehyde (CFA), a food flavoring and agonist of NF-E2-related factor 2 (Nrf2), was selected by multimodal *in vitro* screening, followed by investigation of several downstream effects potentially involved. Furthermore, in the APP/PS1 AD mouse model, the therapeutic effects of CFA (0.2 mmol kg^-1^d^-1^) were tested beginning at 3 months of age. Behavioral phenotypes related to learning and memory capacity, brain pathology and biochemistry, including Aβ transport, were assessed at different time intervals.

**Results**: CFA promoted neuron viability and showed potent neuroprotective effects, especially on mitochondrial structure and functions. In addition, CFA greatly enhanced the brain clearance of Aβ in both free and extracellular vesicle (EV)-contained Aβ forms. In the APP/PS1 mouse model, CFA effectively abolished brain Aβ deposits and reduced the level of toxic soluble Aβ peptides, thus eliminating AD-like pathological changes in the hippocampus and cerebral cortex and preserving learning and memory capacity of the mice.

**Conclusion**: The experimental evidence overall indicated that Nrf2 activation may contribute to the potent anti-AD effects of CFA. With an excellent safety profile, further clinical investigation of coniferaldehyde might bring hope for AD prevention/therapy.

## Introduction

The escalating prevalence of Alzheimer's disease (AD) presents an enormous world-wide challenge to patients, as well as a significant social and economic burden 1. However, to date, a variety of efforts for disease-modifying therapy have failed 2, 3. Most therapies are based on the Aβ-Tau cascade hypothesis, the leading theory to explain AD pathophysiology 4, 5. However, Aβ accumulation occurs over a long period, beginning as much as 10-20 years before the appearance of clinical symptoms 6. Thus, treatment at symptomatic stages might be too late because irreversible damage to neurons has already occurred 7, 8. Hence, treating AD at early stages and targeting processes upstream of AD pathogenesis, such as amyloid β (Aβ) deposition to prevent the onset of AD pathology in the brain, might be a more desirable strategy 8-12.

Because AD pathogenesis is likely dependent on simultaneous dysfunction of multiple pathways 13, 14, identification of therapeutic agents capable of multi-modal targeting of disparate processes in parallel, especially at early stages, might be more efficient in preventing neuronal death and preserving cognitive function. Potential targets include removal of Aβ species, especially the toxic soluble Aβ oligomers 4, 15, 16, from the CNS, a process that is dramatically reduced in AD 17; mitochondrial dynamics and metabolism, which have been suggested as among the earliest manifestations of AD 18, 19; and neurotrophic or similar functions, which when promoted by some compounds can reverse cognitive impairment in AD animals even without reducing the senile plaques 20. Therefore, we sought novel, disease-modifying agents for treatment of AD by simultaneous targeting of multiple pathways, i.e. improvement of neural cell viability and neuroprotection, particularly against mitochondrial dysfunction, from compounds with known safety profiles and promotion of brain Aβ clearance. To achieve this goal, we considered activators of NF-E2-related factor 2 (Nrf2), a master regulator of major cellular defense systems responsible for promoting expression of enzymes that protect against oxidative stress and efflux transporters 21-24. Importantly, inactivation of Nrf2 has been shown to be tightly linked to AD 25, 26. Therefore, we proposed that an effective AD disease-modifying agent may be found by screening Nrf2 agonists.

**Figure 1:**
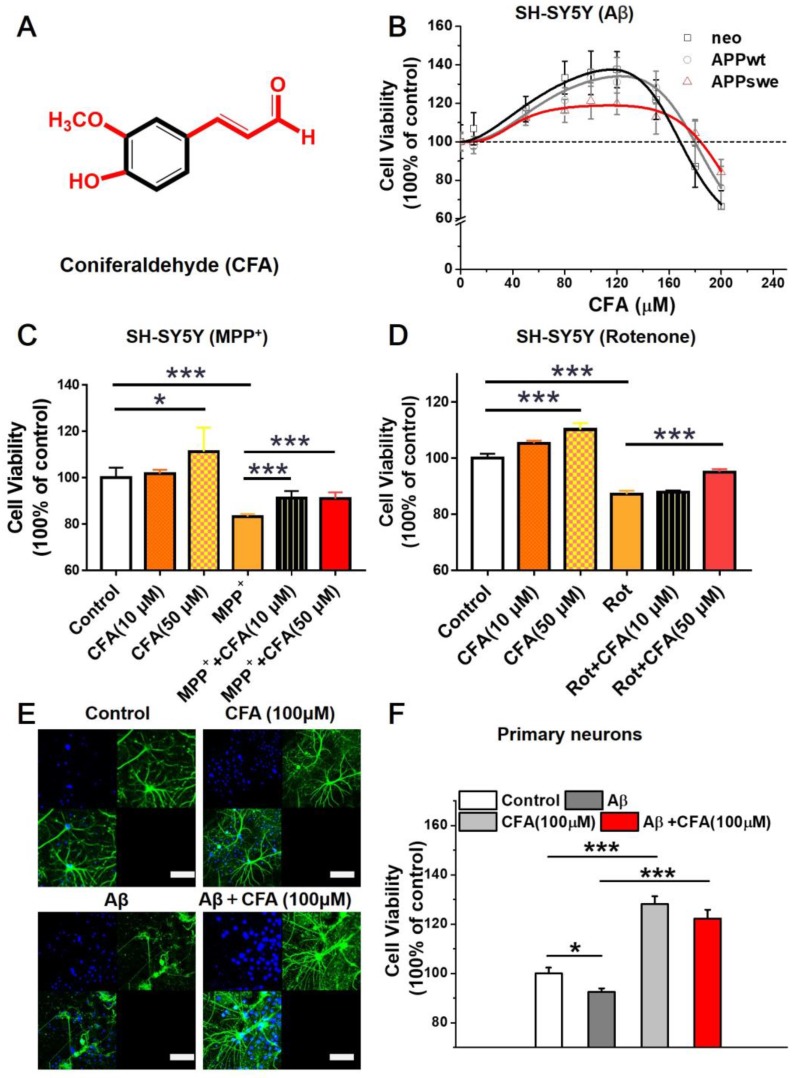
** CFA improved neuronal cell viability and protected cells against Aβ stress and mitochondrial toxins.** (**A**) Chemical structure of CFA. (**B**) The effects of coniferaldehyde (CFA) on the viability of SH-SY5Y cells (neo, APPwt and APPswe) with/without Aβ burden, 300 μM MPP^+^ (1‐methyl‐4‐phenylpyridinium) (**C**) or 1 μM rotenone (Rot) (**D**) 24 h pre-induced SH-SY5Y cells. All kinds of cells were treated with CFA for 36 h before quantification of cell viability by MTS assay. (**E**) Representative immunofluorescence images of CFA-treated and control primary cultured neurons. Mouse primary neurons were treated with 100 μM CFA for 36 h with or without Aβ42 (5 μM) stress. The neurons were visualized with FITC-labeled Microtubule Associated Protein 2 (MAP2) antibodies (green) and Hoechst (blue). Scale bars, 75 μm. (**F**) Improvement of cell viability upon CFA treatment with/without Aβ42 (5 μM) stress in primary cultured neurons. Results are mean±SD (n=3). *P<0.05, ***P<0.001 *versus* control or specific indication.

Based on the activity of cinnamaldehyde, which enhances mitochondrial function while reducing Aβ oligomerization and Tau aggregation 27, 28, we conducted a screening of a variety of cinnamaldehyde analogs, which have the α,β-unsaturated carbonyl group that serves as a potent activator of Nrf2, and identified coniferaldehyde (CFA) (Fig. 1A), a food flavoring 29, 30, that met all of these criteria. CFA effectively promoted neuronal cell viability and provided strong neuroprotective effects to neuronal cells under stress due to elevated levels of Aβ or exposure to mitochondrial toxins, improved mitochondrial function, and significantly enhanced brain excretion of both free and extracellular vesicle (EV)-bound forms of Aβ. Further, CFA gave reassuring results in preservation of learning and memory function in AD model animals and substantial reduction of brain Aβ pathology. Overall, our results suggest CFA is a promising putative preventive/therapeutic agent for AD.

## Materials and Methods

### Materials

Coniferaldehyde (CFA) (98%) and Tretinoin (ATRA) were from Sigma Aldrich Tech Co. (USA). 3-(4,5-dimethylthiazol-2-yl)-5-(3-carboxymethoxyphenyl)-2-(4- sulfophenyl)-2H-tetrazolium (MTS) was from Promega (USA). Arabinoside Cytosine (AraC) and Poly-D-lysine were from Sigma Aldrich Tech Co. (USA). Neurobasal-A medium and Glutamine were from Invitrogen (USA). Minimum Essential Medium Non-Essential Amino Acids (MEM, NEAA) Solution, B-27 and fetal bovine serum (FBS) were from Gibco (USA). Dulbecco's modified Eagle's medium (DMEM) and phosphate buffer saline (PBS) were from Hyclone (USA). Penicillin/streptomycin, MitoTracker Red CMXRos was from Invitrogen (USA). XF Cell Mito Stress Test Kit and XF Glycolysis Stress Test Kit were from Seahorse Bioscience (USA). Reactive Oxygen (ROS) Species Assay Kit and Bicinchoninic Acid (BCA) Protein Quantitation Kit were from Beyotime (China). Mitochondrial Membrane Potential Assay Kit with JC-1 was from Bridgen (China). ATP Bioluminescence Assay Kit was from Beyotime (China). Nrf2 siRNA was from Santa Cruz (USA). Lipofectamine™ 3000 Transfection Reagent was from Thermo Fisher (USA). Primary antibodies: Aβ_1-16_ (6E10) from Biolegend (USA), MAP2, GFAP, Nrf2, HO-1, Drp1, PKM2, p-Tau (ser 262, ser 422), p-GSK-3β (ser 9), p-AKT (ser 473) from Abcam (USA). Goat anti-Rabbit IgG (H+L) Highly Cross-Adsorbed Secondary Antibody (Alexa Fluor 488) was from Abcam (USA). GAPDH and HRPconjugated anti-mouse and anti-rabbit secondary antibodies were from Easybio (China). Dimethylsulfoxide (DMSO) was from Sigma Aldrich Tech Co. (USA). Other reagents were of analytical grade.

### Cell culture and treatment

Three human neuroblastoma SH-SY5Y cell lines (neo, APPwt, and APPswe) were obtained from Institute of Biophysics, Chinese Academy of Sciences; the SH-SY5Y APPwt cells express wild type Aβ precursor protein (APP); SH-SY5Y APPswe cells express APP with the Swedish mutation; SH-SY5Yneo are the blank cells transfected with an empty vector. SH-SY5Yneo cells produce marginal levels of Aβ peptides while the SH-SY5Y APPswe cells generate high concentrations of Aβ up to 1000 pg/ml 31. The cells were cultured in DMEM supplemented with 10% FBS, 1% MEM NEAA, 1% penicillin/streptomycin, 5% CO_2_ atmosphere at 37 °C. These cells were kept selected by G418 resistance.

To observe the effect of CFA on mitochondrial intoxication, SH-SY5Y cells were pretreated with 300 μM MPP^+^ or 1 μM Rotenone for 24 h. The cells were cultured in DMEM/F12 supplemented with 10% FBS, 1% penicillin/streptomycin, 5% CO_2_ atmosphere at 37 °C.

CFA stock solutions were prepared in DMSO, and freshly diluted with culture medium to the working concentrations. After pre-incubation of cells at 37 °C for 24 h, desired concentrations of CFA were added and incubated for 36 h at 37 °C before conducting assays.

### Cell viability

Cell viability was evaluated by MTS assay 32. Briefly, cells (5×10^3^ cells/well) were seeded into 96well plates and incubated for 24 h. Then various concentrations (0.1~200 μM) of CFA were added to wells. After treatment for 36 h, MTS solution diluted with DMEM at a final concentration of 0.2 mg/mL was added and incubated for another 2 h. Finally, the absorbance at 490 nm of each condition was determined on a microplate reader (Thermo Lab systems, Finland).

### Immunofluorescent observation of Nrf2 translocation into the nucleus

The SH-SY5Y cells were grown on 35-mm^2^ confocal dishes (Axygen, USA). After treatment with 100 μM CFA for 36 h at 37 ^o^C, the cells were in turn washed three times with PBS, fixed in 4% formaldehyde for 10-15 min, and made permeable with 1% Triton X-100. Then the cells were blocked in 1% BSA for 30 min. After blocking, the cells were incubated with primary Nrf2 antibody (1:500 dilution in blocking solution) for 3 h at room temperature. After wash, the cells were incubated with fluoresceinisothiocyanate (FITC)-labeled (green) secondary antibodies (1:50) for 2 h at room temperature. The dishes were then counter stained with 40, 6-diamidino- 2-phenylindole (DAPI) for 10 min and covered with 90% glycerol. The fluorescent images were observed on a confocal laser-scanning microscope (Nikon, Japan) with the excitation/emission wavelength at 488/525 nm, and quantified using Image J software.

### Inhibition of Nrf2 with ATRA and small inhibitor RNAs (siRNA)

Inhibition of Nrf2 was conducted using an ATRA concentration that did not significantly affect the cell viability. Briefly, cells (5×10^3^ cells/well) were seeded into 96well plates and incubated for 24 h. Then the cells were treated with 5 μM ATRA for 24 h. After ATRA pretreatment, CFA treatment and following assays were conducted.

Inhibition of Nrf2 was also conducted using an Nrf2 siRNA (Santa Cruz). Briefly, cells were seeded into 6-well or 96well plates and incubated for 24 h. Then the cells were transformed with Nrf2 siRNA for 24 h. After Nrf2 siRNA pretreatment, CFA treatment and following assays were conducted as above.

### Mitochondrial labeling and confocal microscopic observation

The SH-SY5Y cells were grown on 35-mm^2^ confocal dishes (Axygen, USA). After treatment with 10 or 100 μM CFA for 36 h, the cells were incubated with 50 nM MitoTracker Red CMXRos for 10 min at 37 °C. Then the cells were washed three times with FBS-free DMEM medium and observed on a confocal laser scanning microscope (Nikon, Japan) with the excitation/emission wavelength at 579/599 nm. The length of mitochondrial in SH-SY5Y cells was analyzed by an Image J software.

### Determination of intracellular ATP level

The ATP level in SH-SY5Y cells was determined using an ATP bioluminescence assay kit (Beyotime). Briefly, The SH-SY5Y cells were grown on 6-well culture dishes. After CFA (100 μM) treatment, the cells were harvested and lysed with a lysis buffer, followed by centrifugation at 10,000 ×g for 2 min at 4°C. Finally, the level of ATP was determined by mixing 50 μL of the supernatant with equal volume of luciferase reagent, which catalyzed the light production from ATP and luciferin. The emitted light was linearly related to the ATP concentration and measured using a microplate Luminometer (Centro XS^3^ LB 960).

### Measurement of oxygen consumption rate (OCR) and extracellular acidification rate (ECAR)

The mitochondrial OXPHOS function and glycolysis capacity of SH-SY5Y cells were measured by determining the oxygen consumption rate (OCR) and extracellular acidification rate (ECAR), respectively, with a Seahorse XF24 extracellular flux analyzer (USA) 33. SH-SY5Y cells were seeded at 4×10^4^/well and incubated overnight and applied to Seahorse analyzer. Each analysis was conducted in three replicates.

For measuring OCR with intact cells, the experiment was performed in medium consisting of 25 mM glucose, 2 mM sodium pyruvate in unbuffered DMEM, pH 7.4, at 37 °C. The OCR values (pmol/min) were monitored during sequential addition of Oligomycin A (OM; an ATP synthase inhibitor; final concentration, 1 μM), carbonylcyanide *m*-chlorophenylhydrazone (FCCP; a mitochondrial uncoupler; final concentration, 500 nM), antimycin A (AA; complex III inhibitor; final concentration, 1 μM) and rotenone (Rot; complex I inhibitor; final concentration, 1 μM) according to the OCR testing protocol.

For measuring ECAR, the experiments were performed in unbuffered DMEM, pH 7.4, at 37 °C. The ECAR values (mpH/min) were monitored during sequential addition of glucose (final concentration, 10 mM), Oligomycin A (final concentration, 1 μM), and 2-deoxyglucose (2-DG; inhibitor of glycolysis; final concentration, 100 mM) according to the ECAR testing program.

### Primary neuron culture

Culture plates were prepared by coating with poly-D-lysine (40 μg/ml) incubating at 37 °C /5% CO_2_ incubator overnight. The dishes/coverslips were washed three times with sterile water 1-2 hours before beginning the dissection. C57 neonatal mice were prepared by disinfecting their skin with 75% alcohol, removing the whole brain and placing in a 10 cm dish with cold dissection media (50 ml Neurobasal A, 1 ml B-27, 125 μl glutamine). After removal of the meninges and blood vessels under the dissecting microscope, the cortical/hippocampal region was dissected and placed in new cold dissection media. Dissection media was replaced with room temperature (RT) dissection media and incubated at 30 °C for 8 min. Tissue was transferred to enzyme solution (12 ml DMEM, 30 μl glutamine, 48 μl DNAse, 216 μl papain) and incubated at 30 °C for 15 min. Afterwards, tissue was washed with culture media (DMEM 45 ml, FBS 5 ml and 0.5 ml penicillin/streptomycin) three times before two rounds of trituration in culture media. Supernatant was transferred to a new tube through a filter, and centrifuged at 1100 rpm for 5 min in a 50 ml conical tube. Cells were seeded into 96well plates, 6-well plates and other culture plates at a density of 2.5-3 million cells/ml. Media was replaced with Neurobasal Media (47.5 ml Neurobasal A, 1ml B-27, 0.5 ml glutamine, 0.5 ml penicillin/streptomycin) on the following day, then exchanged with fresh media containing AraC at a final concentration of 5 μM. Cells were then cultured for approximately 12 days, with a 1/2 media change every 3 days with Neurobasal Media continuing 5 μM AraC.

Mouse primary neurons were treated with 100 μM CFA for 36 h with or without Aβ_42_ (5 μM) stress. Cell viability was evaluated by MTS assay. Immunofluorescence in primary cultured neurons under CFA protection was visualized with FITC-labeled MAP2 antibodies (green) and Hoechst (blue) on a confocal laser-scanning microscope (Nikon, Japan) with the excitation/emission wavelength at 488/525 nm.

### Mice

APPswe/PS1dE9 (APP/PS1) transgenic mice (male, female, SPF grade) and littermate negative C57BL/6 mice (male, SPF grade) were purchased from Model Animal Research Center of Nanjing University. The APP/PS1 mice over-express the delta exon 9 variant of presenilin 1 (PS1) in combination with the Swedish mutation of β-amyloid precursor (APP). The mice were maintained and handled with the approval of Institutional Review Board for Laboratory Animal Care (Approval No. LA2015054), and fed in a barrier environment in Department of Laboratory Animal Science, Peking University Health Science Center.

Mice were allocated randomly into four groups as follows: (I) WT (littermate negative C57BL/6 male mice) (n=8~10) as the negative control; (II) APP/PS1 (untreated mice) (n=8~10) as the positive control; (III) ineffective low dose (0.02 mmol kg^-1^day^-1^) CFA-treated APP/PS1 mice (n=8~10); (IV) effective high dose (0.2 mmol kg^-1^day^-1^) CFA-treated APP/PS1 mice (n=8~10). The drug administration started at 3 months of age, by feeding the animal with pellet food containing desired amounts of CFA. Throughout the experimental period, the body weight, water and food intake were monitored. The behavior evaluations including step-down passive avoidance tests and Morris water maze (MWM) experiments were performed to monitor the alteration of learning and memory ability.

### Step-down passive avoidance test

At 5 months old (2 months after drug oral administration), the learning and memory in the mice were examined by using the step-down type of passive avoidance task. The test apparatus consisted of eight chambers (12×12×18 cm) having a grid floor with a wooden platform (5×5×4.5 cm) at the right lower corner of the grid floor within a chamber illuminated with a 15 W lamp. During the experimental period, each mouse was gently placed on the grid floor with back against the platform, and electric shocks (0.25 mA) were continually delivered to the grid floor. When the mice receive electric stimulus, they find and jump up on the platform; however, the mice will jump down when they forget the electric shock. The time period for mice staying on the platform between a jump-up and jump-down was recorded as step-down latency. The number of step-down events during the 5-min trial was counted as “errors”. The first step-down latency and error are regarded as the learning latency and learning error, respectively. The tests were repeated after different retention times (24 h and 1 month), and the corresponding data were regarded as memory latency and memory error.

### Morris water maze test

The Morris water maze (MWM) task was performed at the end of the drug treatment. The maze consisted of a circular pool (150 cm in diameter, 60 cm in height) filled with water (temperature at 22 ± 1 °C), which was rendered opaque by adding white food dye. The pool contained various prominent visual cues and conceptually divided into four equal quadrants (I, II, III, IV) by imaging lines. The performance of the mice was recorded with an automated tracking system during all phases of the task.

*Visual cue phase.* Following habituation, visible platform training was performed to measure motivation of the mice to find a platform, visual acuity of the mice, and the ability of mice to use local cues. On day 1-3, a platform (diameter, 10 cm), half-way between the center and the wall, was labeled with a flag and placed 1 cm above the surface of the water in the center of a quadrant. All animals entered the water facing the wall of the pool and were allowed to explore the platform for 60 s, and if they reached the visible platform, they remained there for 5 s. If they did not find the platform within 60 s, they were guided there by the experimenter and remained there for 20 s. The starting quadrant was changed to the II, III, IV quadrants respectively each day. During the visual cue phase of training, speed and latency to the platform were used to compare the performance between animal groups and screen out mice with poor eyesight.

*Acquisition phase.* On day 4-9, the last hidden platform trial was conducted to measure the ability of mice to form a representation of the spatial relationship between a safe, but invisible (submerged 1 cm below the water level) platform (10 cm in diameter) without flag and visual cues surrounding the maze. The platform was replaced in the center of IV quadrant, and several extra maze cues were distributed across the walls surrounding the pool. The starting quadrant was changed in I, II, III quadrants respectively each day. Animals were allowed 60 s to locate the platform and 5 s to rest on it. Mice that failed to find the platform were led there by the experimenter and allowed to rest there for 20 s. The acquisition time (latency to reach the platform) and path length (distance swam to the platform) were used to analyze and compare the performance between different treatment groups.

*Probe trial phase.* Twenty-four hours following the last hidden platform trial, the probe test was carried out by removing the platform and allowing each mouse to swim freely for 60 s. All animals were placed in quadrant I, opposite the target quadrant IV (where the platform had been located during hidden platform training). The time and distance that mice spent swimming in the four quadrants and other parameters were recorded to assess the ability of the mouse to remember the previous location of the platform.

### Mouse brain samples

One week after the Morris water maze (MWM) task, all of the animals were weighed and euthanized. The brain was immediately harvested and separated sagitally in two hemispheres. One hemibrain was fixed overnight by immersion in 10% buffered formalin at RT and embedded in paraffin. The other hemibrain was dissected on ice to obtain hippocampus, frontal lobe, and cerebellum, and samples were stored at -80 °C for further analysis.

### Human brain samples

Sections of cortex were obtained from the brains of healthy aged control and Alzheimer's disease patients at autopsy following protocols approved by the University of Washington School of Medicine. In all cases where AD was diagnosed at autopsy, AD was stated as the cause of death. AD subjects had no evidence of other neurological disease based on neuropathological examination.

### Brain histology and immunohistochemistry

The 5-mm-thick paraffin sections of hemibrain were prepared for haematoxylin-eosin (HE) staining, Nissl staining and amyloid-β (Aβ) immunohistochemistry. Quantitative analysis of surviving cells was performed at 40× magnification (Olympus BX51, JAPAN). The total number of neurons without basophilic lesions in the dentate gyrus, as well as the total number of neurons and basophilic cells in combined CA1-4, were counted in HE pathological sections. Mean values were calculated from 5-6 fields in three levels with HE pathological sections per animal in each region. Immunostaining of Aβ forms was performed using a 6E10 antibody (1 : 200; BioLegend) by overnight incubation at 4 °C. The HRP-conjugated secondary antibody was then incubated for 1 h at room temperature. The images were visualized finally with DAB (3,3-diaminobenzidine), paying special attention to the hippocampus, cerebral cortex and cerebellum.

The Nrf2 immunohistochemistry was conducted the same as above.

### Brain tissue sequential extraction

All extractions were performed on ice or at 4 ^o^C. Weighed samples of hippocampus, cerebral cortex and cerebellum tissues were added to 10 times the mass of pre-chilled Tris buffer (1 mM Tris, 1 mM EGTA, 1 mM DTT, 10% sucrose, pH 7.5) and homogenized with a bullet blender (Gene Company Limited, Hong Kong). Then homogenates were centrifuged at 26,000×g for 15 min. The supernatant (**water soluble fraction**) was then collected and stored at -80°C. The pellet was re-suspend in pre-chilled 1% Triton X-100 solution containing 1 mM Tris-HCl (pH 7.5), 1 mM EGTA, 1 mM DTT, and 10% sucrose. Then, the sample was centrifuged at 23,0000×g for 1 h. The supernatant (**water insoluble/lipid soluble fraction**) was collected and stored at -80°C.

### Preparation of Eu-DTPA-Aβ

Eu-DTPA-Aβ was prepared according to the previous method 34. Briefly, the DTPA-Aβ conjugates were prepared by adding 0.18 mg of DTPAA dissolved in DMSO to Aβ (1 mg) solution in 1ml 0.05 M carbonate buffer solution (pH 8-10) under vigorous stirring. The coupling reaction proceeded 3-4 h at r.t. in order to make Aβ react completely 35. Then, the reaction solution was dialyzed (cut off 1kDa) by phosphate buffered solution (pH 7.0) to remove the unreacted DTPAA. Then, 0.01M EuCl_3_ was added dropwise until appearance of a white sediment to prepare the Eu-DTPA-Aβ. After centrifugation (3 min, 10000×g ), the supernatant (Eu-DTPA-Aβ) was collected and applied to a PD-10 desalting column (GE Health Care, USA), which was pre-balanced with HBSS (pH 7.0). The elute was concentrated by centrifugal ultrafiltration (Amicon Ultra-4, cut off 3 kDa). The amount of Aβ was measured with enhanced BCA protein assay kit. The bound Eu was measured by time-resolved fluorescence as described in the previous method (fluorescent parameter: λ_ex/em_ 340/616 nm; measurement window, 600-1000 μs).

### Intrastriate Eu-DTPA-Aβ injection

APPswe/PS1dE9 (APP/PS1) transgenic mice (male, SPF grade) and littermate negative C57BL/6mice (male, SPF grade) were purchased from Model Animal Research Center of Nanjing University. Mice were allocated randomly into three groups as follows: (I) WT (littermate negative C57BL/6 male mice) (n=4~6) as the negative control; (II) APP/PS1 (untreated mice) (n=4~6) as the positive control; (III) CFA-treated APP/PS1 mice (0.2 mmol kg^-1^day^-1^) (n=4~6). The drug administration started at 5 months of age, by feeding the animal with pellet food containing desired amounts of CFA. 2-3 weeks after drug oral administration, the Aβ brain clearance experiments in the mice were conducted.

Anesthetized mice were fixed in a stereotaxic frame and body temperature was kept at 37 ºC with a temperature-controlled warming pad. A 30 GA needle was inserted *via* a small burr hole into the brain at the following coordinates: intra-striate injections (0.22 mm caudal, 2.5 mm lateral, 3.5 mm ventral to bregma). After needle insertion, 30 minutes was allowed to elapse to allow the needle track to swell closed. 2.0 µl of Eu-DTPA-Aβ (dissolved in artificial CSF) was injected at a rate of 0.2 µl/min with a syringe pump (Harvard Apparatus).

After 15, 30 or 60 minutes, animals were rapidly decapitated and the blood was collected, then the skull opened, the dura removed and the brain harvested. The brain was solublized and ground in H_2_O at 4 ºC. Brain fluorescence was normalized to total fluorescence detected in a 2 µl Eu-DTPA-Aβ put directly into a fresh brain to grinder and expressed as the % of total injected fluorescence. Eu-DTPA-Aβ clearance from the brain and accumulation in the blood was compared by two-way ANOVA.

The EVs were extracted by XYCQ EV Enrichment KIT. And the bound Eu was measured by time-resolved fluorescence as described in the previous method.

### Intracisternal FITC-Aβ injection and *in vivo* fluorescence imaging

A craniotomy (2×2 mm in diameter) was made over the cortex of the anesthetized mice. The dura was left intact and the craniotomy was covered with ACSF and sealed with a glass coverslip. Then anesthetized mice were fixed in a stereotaxic frame and a 30 GA needle was inserted into the cisterna magna. 2 µl of CSF FITC-Aβ tracer was injected at a rate of 0.2 µl/min over 10 minutes with a syringe pump (Harvard Apparatus). To visualize the vasculature, 0.1ml BBB impermeable Cy5-dextran 70 (MW 70 kD; both 1% in saline, Nanocs) was injected intra-arterially immediately before imaging. A 20× (0.9 NA) water immersion lens was used to image the cortex, from the surface to a depth of ~240 µm. The cerebral vasculature was first imaged with 512×512 pixel frames from the surface to a depth of 240 µm with 3 µm z-steps. After intracisternal injection of CSF tracer, tracer movement into the cortex was conducted with dual-channel (FITC and Cy5) 512×512 pixel image acquisition.

### Western blotting

The tissue samples were prepared as described above. The cell samples were washed twice with ice-cold phosphate buffer solution (PBS) and lysed on ice with pre-cooled RIPA lysis buffer containing protease and phosphatase inhibitor cocktails. The samples were centrifuged at 12,000×g for 20 min at 4°C. The supernatant was collected.

The total protein levels of the samples were determined with a BCA protein quantitation kit. Aliquots containing 25 μg total protein were applied to SDS-PAGE gel separation. Then, the protein bands were transferred to polyvinylidene fluoride (PVDF, Millipore) membranes. After blocking with 5% nonfat dry milk or 5% (Bovine Serum Albumin) BSA for 2 h, membranes were incubated with desired primary antibodies (Aβ, Nrf2, HO-1, Drp1, PKM2, p-Tau, p-GSK-3β, p-AKT or GAPDH) overnight at 4°C and then secondary antibody for another 1h at room temperature. After washes, the protein bands were visualized with an ECL Western blotting substrate kit and observed on a chemi-luminescence imaging system. The protein bands were quantified using ImageJ software (National Institutes of Health, USA).

### Statistical analysis

Data were expressed as mean±standard deviation (SD) or mean±standard Error of Mean (SE). Differences between groups were analyzed by two-way analysis of variance (ANOVA) and *P*<0.05 was considered as statistically significant. Statistical analyses were carried out using Origin 8.0 or SPSS.

## Results

### Coniferaldehyde promotes neural cell viability and protects cells from Aβ stress and mitochondrial toxins

We screened a variety of cinnamaldehyde derivatives including *o* or *m* or *p*-hydroxycinnamaldehyde, *o* or *m* or *p*-methoxycinnamaldehydes, ferulaldehyde, sinapaldehyde, and 4-hydroxy-3-methoxycinnamaldehyde (coniferaldehyde, CFA) in three SH-SY5Y cellular models stably transformed with a control vector (neo), a vector expressing amyloid precursor wild type protein (APPwt), or a vector expressing APP protein with the Swedish mutation (APPswe), respectively. Among the tested compounds, CFA best promoted cell growth in all three cellular models. The effects were significant within the range of 30~160 μM, with maximal promotion at 80~100 μM (Fig. 1B). It is noted that CFA treatment significantly up-regulated the level of brain-derived neurotrophic factor (BDNF) (Fig. S1), suggesting the possible involvement of neurotrophic signaling pathways in the actions of CFA.

Because cinnamaldehyde has previously demonstrated mitochondrial protection against oxidative stress 30, the ability of CFA to reduce damage induced by the mitochondrial-targeting agents MPP^+^ (1-methyl-4-phenylpyridinium) and rotenone (Rot) was examined. CFA (50 μM) effectively alleviated the neuronal cell damage (Fig. 1C-D) caused by MPP^+^ or Rot. Even 10 μM CFA significantly reduced SH-SY5Y cell death induced by MPP^+^ treatment (Fig. 1C). These results indicate that CFA protects neural cells, likely *via* mitochondria.

To further characterize the neuroprotection by CFA, primary cultured neurons were stressed by exposure to 5 µM Aβ_42_ and then treated with 100 µM CFA. Neurons exposed to Aβ_42_ demonstrated morphological disruption including reduction of axon outgrowth and decreased neurite elaboration (Fig. 1E-F). Remarkably, CFA treatment significantly preserved the normal morphology of primary neurons (Fig. 1E) and improved cell viability (Fig. 1F) in both the absence and presence of Aβ_42_. All the results above indicated that CFA is an excellent neuroprotective agent.

### The role of Nrf2 signaling in the protective effect of coniferaldehyde

**Figure 2:**
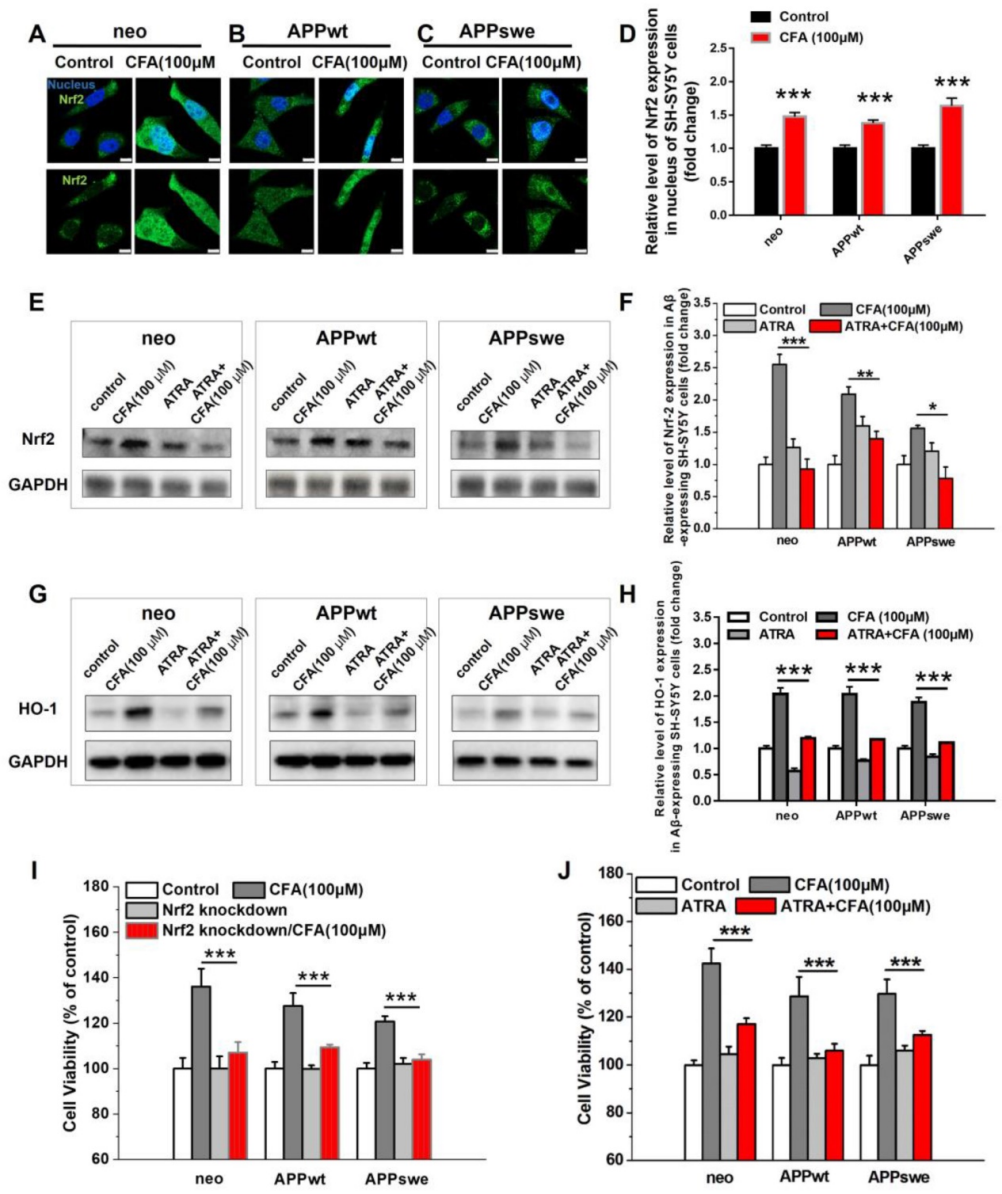
** CFA treatment activated Nrf2 in SH-SY5Y cells with & without Aβ burden responsible for most neuronal protective effects. (A-C)** Representative immunofluorescence image of Nrf2 (green) translocation into nucleus (blue) upon treatment of Aβ-expressing SH-SY5Y (neo, APPwt and APPswe) cells with 100 μM CFA. Scale bars, 10 μm. **(D)** Quantification of nuclear *vs* cytoplasmic Nrf2 expression based on the imaging (A-C). (**E**-**H**) Nrf2 expression (**E**,**F**) and HO-1 expression (**G**,**H**) were elevated upon CFA treatment (100 μM) but inhibited by pretreatment with all-trans-retinoic acid (ATRA, 5 μM, 24 h); (**I**) Nrf2 knockdown with siRNA significantly decreased improvement of cell viability by CFA (100 μM). (**J**) ATRA treatment significantly decreased improvement of cell viability by CFA (100 μM). Results are mean±SE (*n*=3). *P<0.05, **P<0.01, ***P<0.001 *versus* CFA group.

We next sought to confirm that CFA protects neurons through activation of the Nrf2 pathway, as hypothesized based on our screening process. Under the resting state, Nrf2 is sequestered in the cytoplasm by its binding partner, Kelch-like ECH-associated protein 1 (Keap1) 21-24, 36, 37. Upon activation, it is translocated to the nucleus, where it activates its downstream targets. Therefore, we investigated the localization of Nrf2 following CFA treatment. As expected, CFA treatment significantly promoted the translocation of Nrf2 from the cytosol to the nucleus (Fig. 2A-D and S2). While Nrf2 levels were lower in Aβ-overexpressing cells (APPwt and APPswe) (Fig. S3) than in SH-SY5Y neo cells, CFA treatment dose-dependently increased Nrf2 expression in all three cell lines (Fig. 2E-F and S3) as well as up-regulated the expression of HO-1 (Fig. 2G-H and S3), a major downstream target of Nrf2 activation 38. Overall, this evidence demonstrates activation of Nrf2 by CFA *in vitro*.

To further demonstrate whether the neuroprotective and survival-enhancing effects of CFA are mediated by Nrf2 activation, the cells were pretreated with Nrf2 siRNA to knock down expression 39 or all-trans-retinoic acid (ATRA) to block Nrf2 signaling 40. The conditions for Nrf2 knockdown were carefully selected to reduce Nrf2 levels (Fig. S4) without significantly affecting the cell viability. The results (Fig. 2I) showed that Nrf2 knockdown mostly diminished CFA-induced increase of cell viability (by 60~80%) in the three SH-SY5Y cells. Similarly, inhibition of Nrf2 signaling with ATRA also largely reduced CFA-induced increase in cell viability (by 60~70%) in the SH-SY5Y cells (Fig. 2J) and abolished HO-1 expression by CFA (Fig. 2G-H). All these results indicated Nrf2 activation is responsible for most neuroprotective effects of CFA against Aβ toxicity.

### Coniferaldehyde protects mitochondrial structure and function in SH-SY5Y cells with Aβ burden

Aβ-induced mitochondrial dysfunction has been shown to be tightly linked to AD development and progression 18, 19, 41. The mitochondrial dysfunction is mainly a shift of mitochondrial dynamics towards fission 42-44 and of energy metabolism from primarily driven by oxidative phosphorylation towards glycolysis (similar to the Warburg effect 45, 46 in cancer). Therefore, we tested the effects of CFA on mitochondrial structure and function in SH-SY5Y cells.

**Figure 3:**
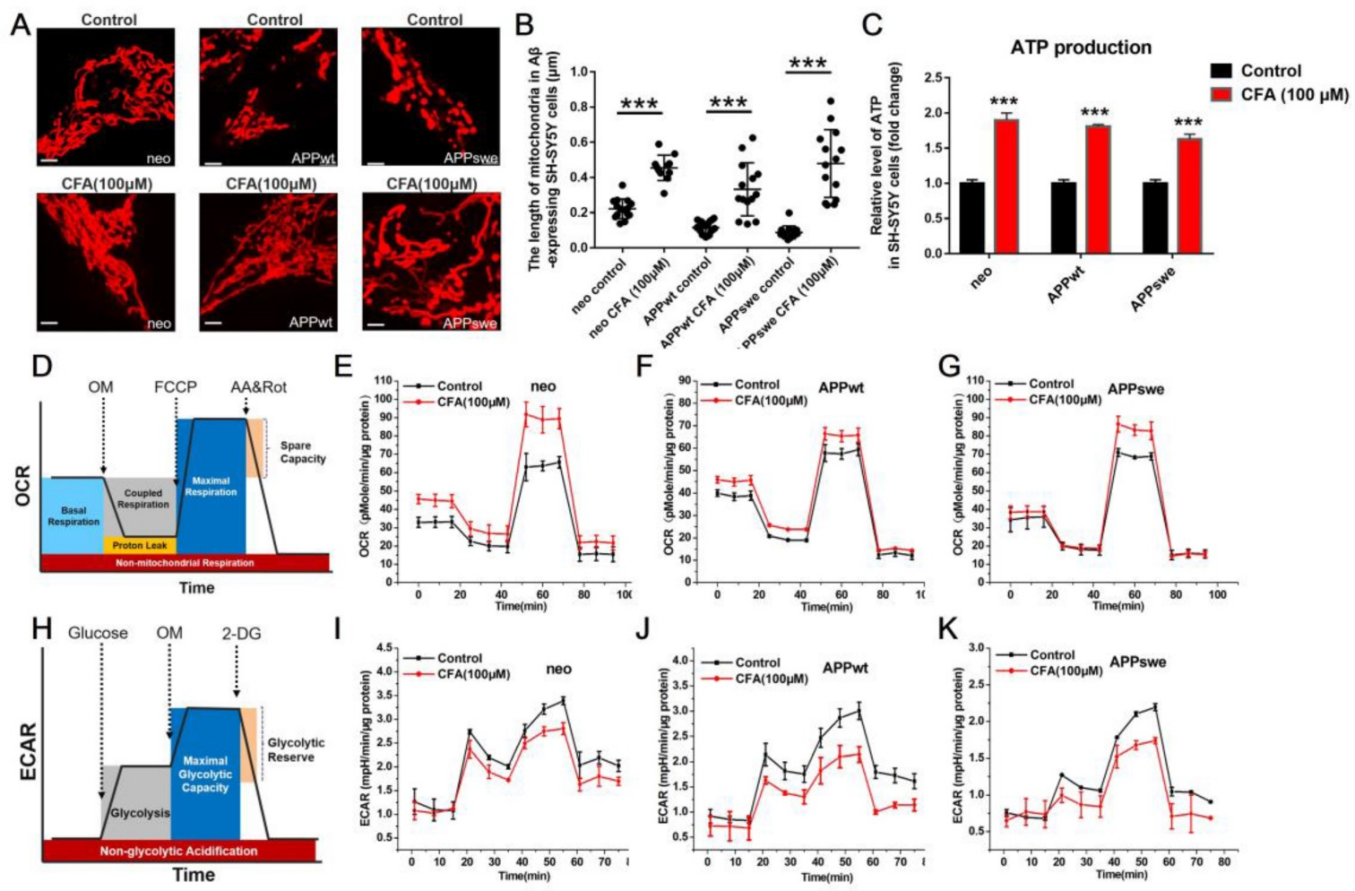
** CFA treatment improved the mitochondrial morphology and energy production in Aβ-expressing SH-SY5Y cells.** (**A-B**) Morphological changes of mitochondria in SH-SY5Y cells with/without Aβ burden upon CFA treatment. SH-SY5Y cells (neo, APPwt and APPswe) were treated with 100 μM CFA and mitochondria were stained with MitoTracker Red CMXRos. Scale bars, 2.5 μm. (**C**) Substantial increase in ATP production by CFA treatment. (**D**-**K**) Enhancement of mitochondrial aerobic respiration/oxidative phosphorylation by CFA. **D,H**: illustration of oxygen consumption rate (OCR) and extracellular acidification rate (ECAR) measurement on a Seahorse XF Extracellular Flux Analyzer, in which oligomycin (OM) inhibits ATP synthase to block coupled respiration, FCCP serves as a mitochondrial uncoupler to resume oxygen consumption, AA (a complex III inhibitor) and Rot (complex I inhibitor) halt oxidative phosphorylation, and 2-deoxyglucose (2-DG) inhibits hexokinase to stop glycolysis. As Figure 3H-K showed, CFA stimulated a slight decrease in ECAR over basal glycolysis, but result in an obvious decrease in maximum glycolytic capacity; **E-G**: The OCR of Aβ-expressing SH-SY5Y cells upon 100 μM CFA treatment; **I-K**: The ECAR of Aβ-expressing SH-SY5Y cells upon 100 μM CFA treatment. Each experimental group was analyzed using three replicates in each analysis. Results are mean±SD (*n*=3).

First, we examined the effect of CFA on mitochondrial morphology. As shown in Fig. 3A, mitochondria in the SH-SY5Yneo cells had normal, threadlike shapes. However, most mitochondria in the APPswe cells were swollen and oval-shaped, with an intermediate phenotype in APPwt cells. Upon treatment with CFA (100 μM), most mitochondria in the APPswe cells regained normal thread-like morphology. To our best knowledge, CFA is the first agent that could reverse the mitochondria fission (Fig. 3A-B) induced by Aβ burden.

Next, we investigated the effect of CFA treatment on ATP production in all three cells using a bioluminescence-based assay, and found that CFA treatment significantly increased it in all three cell lines (Fig. 3C). We further measured the contribution of the two major aspects of mitochondrial ATP production 47, i.e. oxidative phosphorylation and glycolysis, on a Seahorse XF Extracellular Flux Analyzer. In the tests of oxygen consumption rate (OCR), a measure of oxidative phosphorylation (Fig. 3D), CFA increased the basal and maximal respiration rates mostly without affecting the coupled respiration (Fig. 3E-G). In contrast, the extracellular acidification rate (ECAR) (Fig. 3H), largely a measure of glycolysis, was decreased by CFA, suggesting decrease of glycolysis in the three SH-SY5Y cells (Fig. 3I-K). Together, these results indicated that CFA significantly promoted the pattern of ATP production towards oxidative phosphorylation, i.e. improved mitochondrial function.

**Figure 4:**
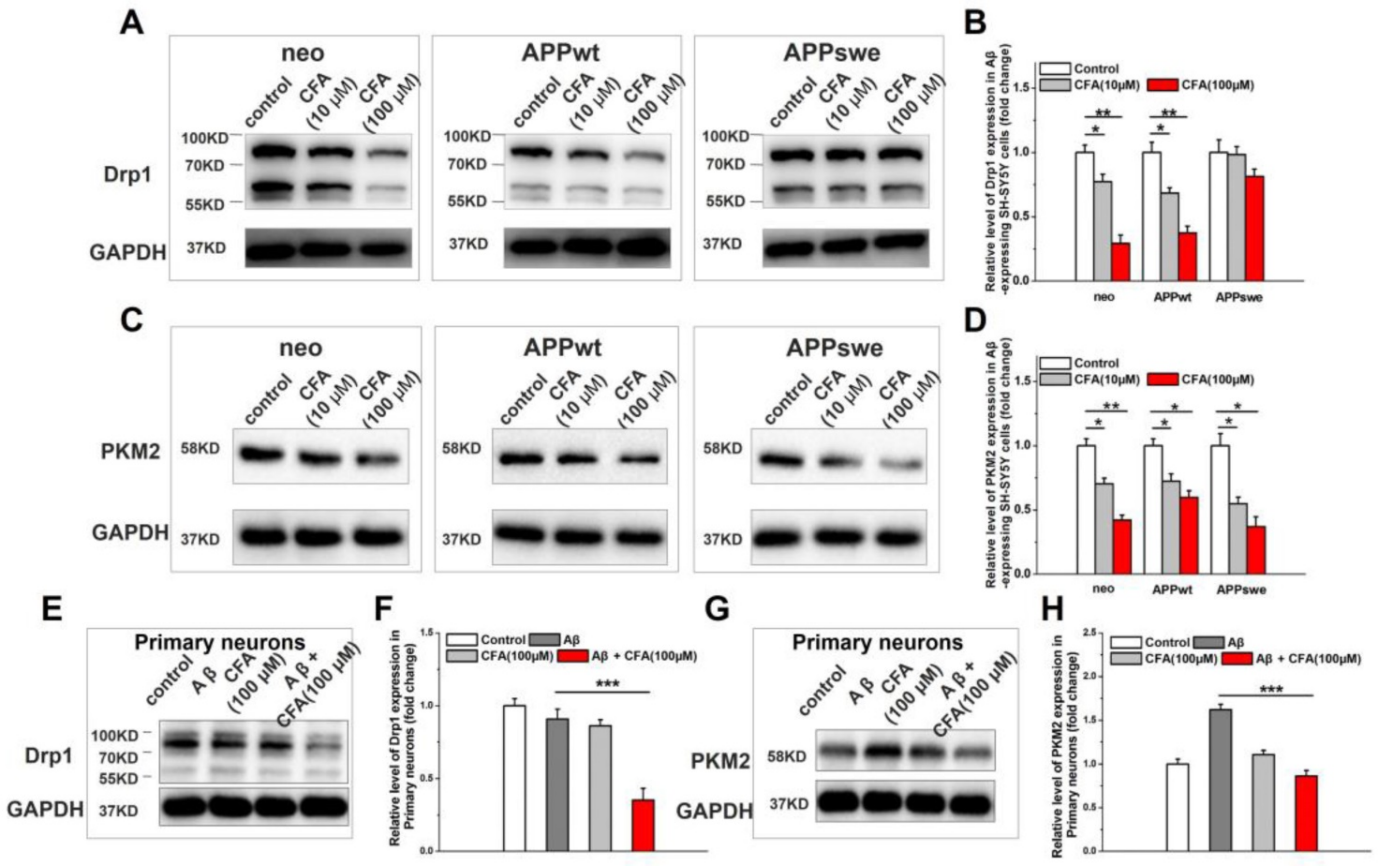
** The effect of CFA treatment on Drp1 expression and PKM2 expression on Aβ-expressing SH-SY5Y cells and primary cultured neurons upon Aβ_42_ treatment.** SH-SY5Y cells (neo, APPwt and APPswe) were treated with 100 μM CFA and the levels of Drp1 (**A**,**B**) and PKM2 (**C**,**D**) were analyzed by western blot. *P<0.05, **P<0.01 *versus* control. Primary cultured neurons were treated with100 μM CFA in presence/absence of 5 μM of Aβ_42_ for 36 h, Drp1 (**E**,**F**) and PKM2 (**G**,**H**) levels were analyzed by western blot. Results are mean±SE (*n*=3). ***P<0.001* versus* Aβ group.

We next explored the effect of CFA on the key proteins regulating mitochondrial structure and function. First, we measured CFA-induced changes on Drp1, a member of the family of large GTPases that stimulates fission when recruited to the mitochondrial membrane 43, 48, 49. As shown in Fig. 4, CFA treatment caused a dose-dependent decrease in Drp1 expression in all SH-SY5Y cell lines (Fig. 4A-B) albeit less significantly in APPswe cells, where the Aβ burden in the highest. Similarly, CFA treatment also reduced Drp1 level in primary cultured neurons under Aβ stress (Fig. 4E-F) and membrane-associated Drp1 in brain of APP/PS1 mice (Fig. S5). Next, we sought to determine the effect of CFA on pyruvate kinase isoform 2 (PKM2), which catalyzes the rate-limiting step of glycolysis and is crucial for the Warburg effect 45, 46. CFA dose-dependently down-regulated PKM2 expression in SH-SY5Y cells (Fig. 4C-D) or abolished Aβ-induced PKM2 elevation in primary cultured neurons (Fig. 4G-H). Together with the improvement of mitochondrial oxidative phosphorylation shown in Fig. 3 above, this finding is consistent with a restoration of normal energy production and a reversal of the Aβ-induced shift toward glycolysis.

In addition, CFA (100 μM) treatment ameliorated dysfunction of mitochondrial membrane potential (ΔΨ_m_) and reactive oxygen species (ROS) production in APPwt and APPswe cells (Fig. S6 and S7). All these results are consistent with a reversal of Aβ-induced shift of mitochondrial energy metabolism.

### The potential effects of CFA on Tau pathology

**Figure 5:**
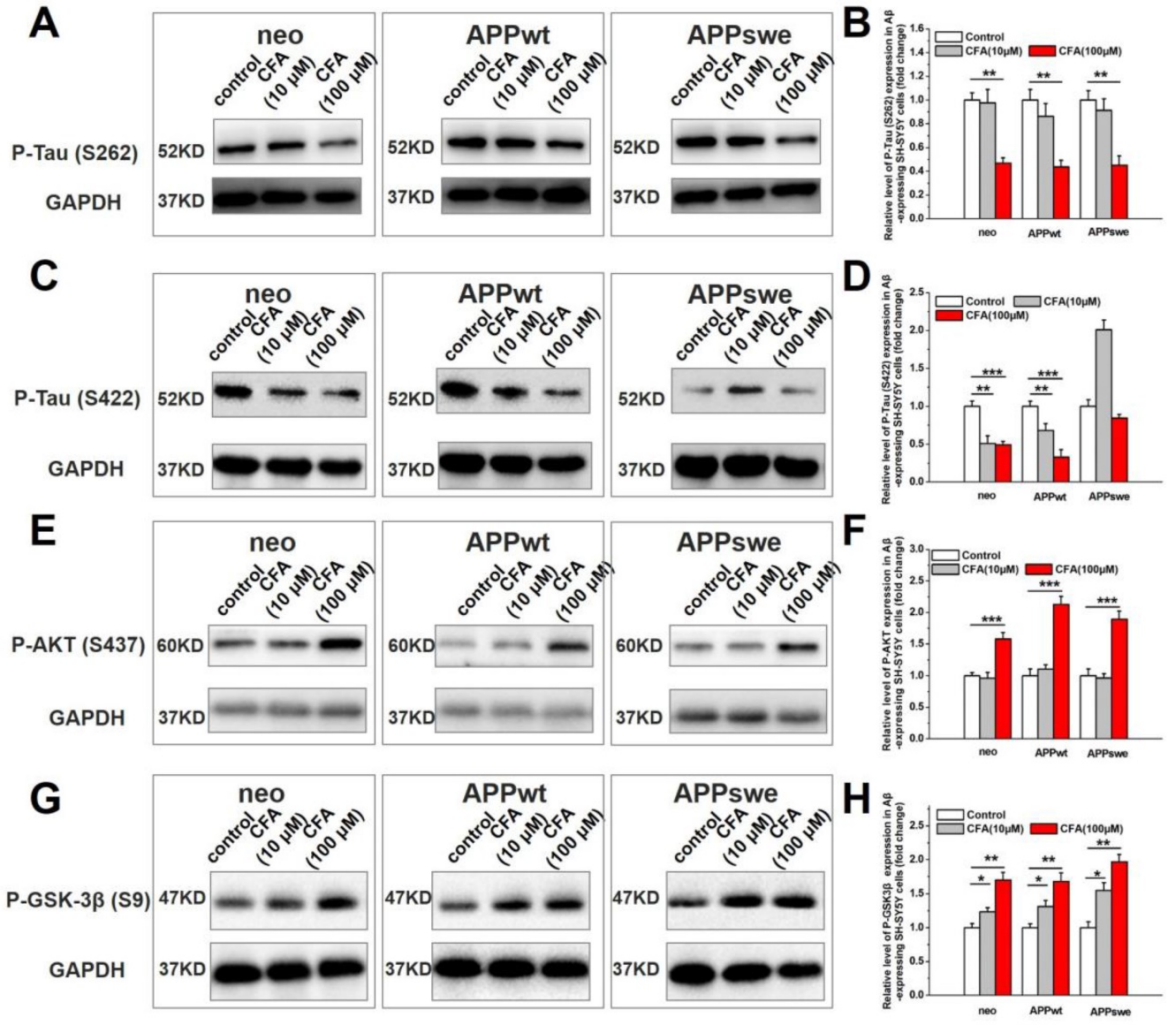
** The effect of CFA on Tau phosphorylation in SH-SY5Y cells**. Western blot analysis of phosphorylation of Tau (S262) (**A,B**), Tau (S422) (**C,D**), Akt (S437) (**E,F**), GSK-3β (S9) (**G,H**). Results are mean±SE (*n*=3). *P<0.05, **P<0.01, ***P<0.001.

In AD pathogenesis, there is crosstalk between Aβ toxicity and intracellular neurofibrillary tangles (NFTs), another histological hallmark of AD 50, 51, which are composed of hyperphosphorylated microtubule associated protein Tau 50, 51. We observed a dose-dependent decrease of p-Tau (S262) (Fig. 5A-B) and p-Tau (S422) (Fig. 5C-D) in Aβ-expressing cells upon CFA treatment. The precise mechanism underlying this phenomenon remains to be investigated; but it is possible that improved mitochondrial energy metabolism increases the phosphorylation-dependent activation of Akt/PKB (Fig. 5E-F) and subsequent phosphorylation inactivation of glycogen synthase kinase-3β (GSK-3β, a major kinase for Tau 52) (Fig. 5G-H). It is also possible that increased Aβ transport (see below for more results and discussion) attenuated Aβ-tau cascade, as proposed by others.

### Coniferaldehyde treatment re-activates Nrf2 in APP/PS1 mice

**Figure 6:**
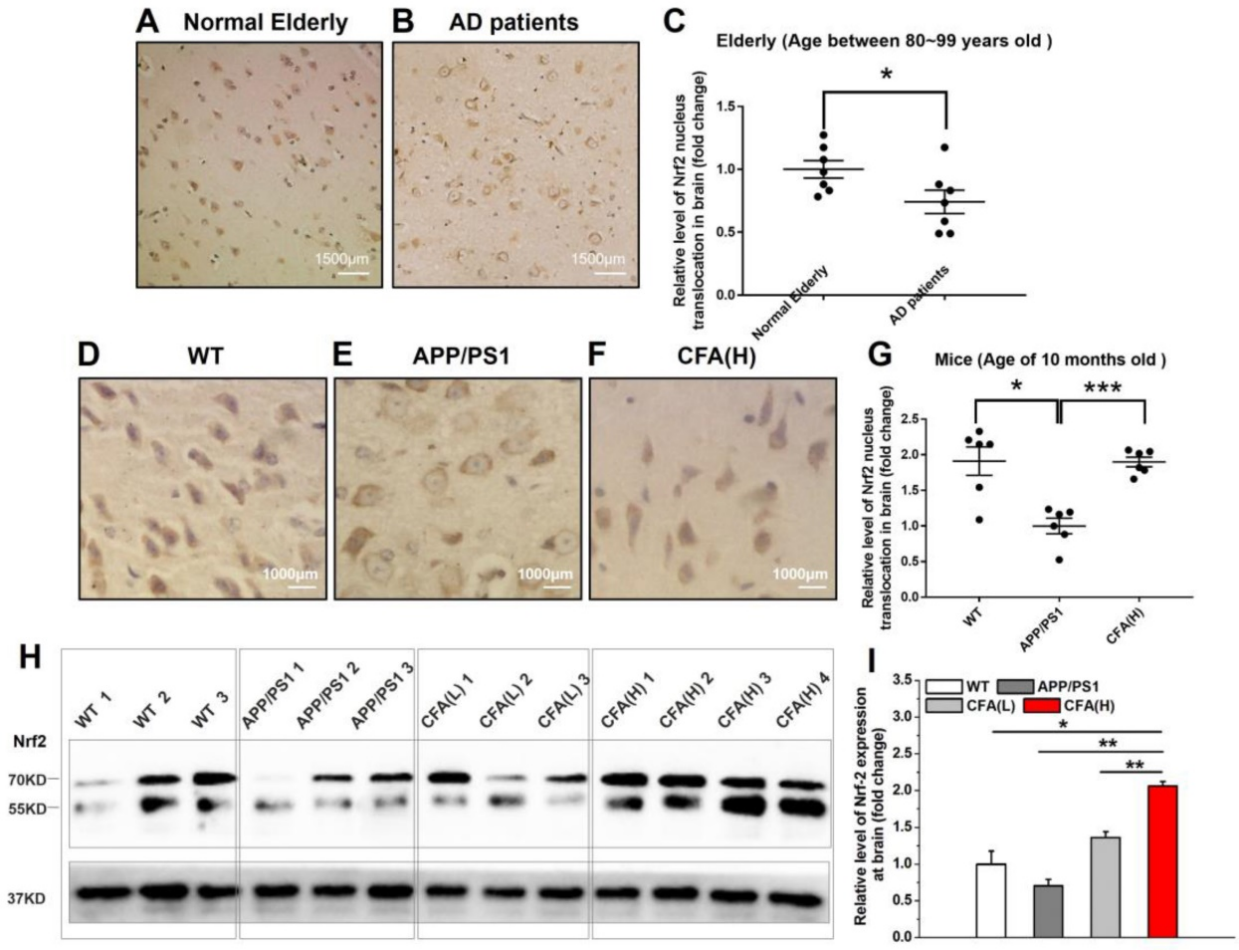
** CFA treatment strongly activated Nrf2 signaling *in vivo*.** The brain samples were immunostained with the Nrf2 antibody. (**A**-**B**) Representative images of Nrf2 staining of brain sections of Normal Elderly controls (**A**) and Alzheimer's Disease patients (**B**). (**C**) Quantification of amounts of nuclear Nrf2 in (**A**-**B**). The mean age of the AD patients was 84.7±2.1 years, and the controls 85.2±3.3. Scale bars, 1500 μm. Results are mean±SE (*n*=7). *P<0.05 *versus* Normal Elderly. (**D**-**F**) Representative images of Nrf2 staining of brain sections of WT mice (**D**), untreated APP/PS1 control (**E**) and CFA(H)-treated (**F**) mice. (**G**) Quantification of amounts of nucleus Nrf2 in (**D**-**F**). Scale bars, 1000 μm. (**H**, **I**) Western blot of Nrf2 levels in brain of WT and APP/PS1 mice with/without CFA treatment. The mice were 10 months old and had been treated with CFA in pellet food for 7 months. Results are mean±SE (n=6, with random selection). *P<0.05. Results are mean±SE (*n*=6, with random selection). *P<0.05, ***P<0.001 *versus* untreated APP/PS1 or specific indication.

In AD patients, Nrf2 was found in the inactive state as indicated by retention of Nrf2 in the cytosol 25, 26. Upon histological analysis of Nrf2 subcellular localization in brains of AD patients obtained at autopsy, we found that the level of nuclear Nrf2 was significantly lower than that of normal elderly controls (Fig. 6A-C). Consistently, a similar pattern of Nrf2 subcellular localization was observed in APP/PS1 AD model mice *versus* WT control (Fig. 6D-G), suggesting chronic Aβ toxicity may be one major reason for Nrf2 inactivation. Not surprisingly, CFA treatment up-regulated Nrf2 expression in a dose-dependent manner (Fig. 6H-I) and particularly, CFA(H) (high dose, 0.2 mmol kg^-1^d^-1^) dramatically reversed the nuclear localization of Nrf2 (Fig. 6F-G), indicating the recovery of Nrf2 activity.

### Coniferaldehyde enhanced brain Aβ excretion *via* both free and extracellular vesicle-bound forms in APP/PS1 AD mice

**Figure 7:**
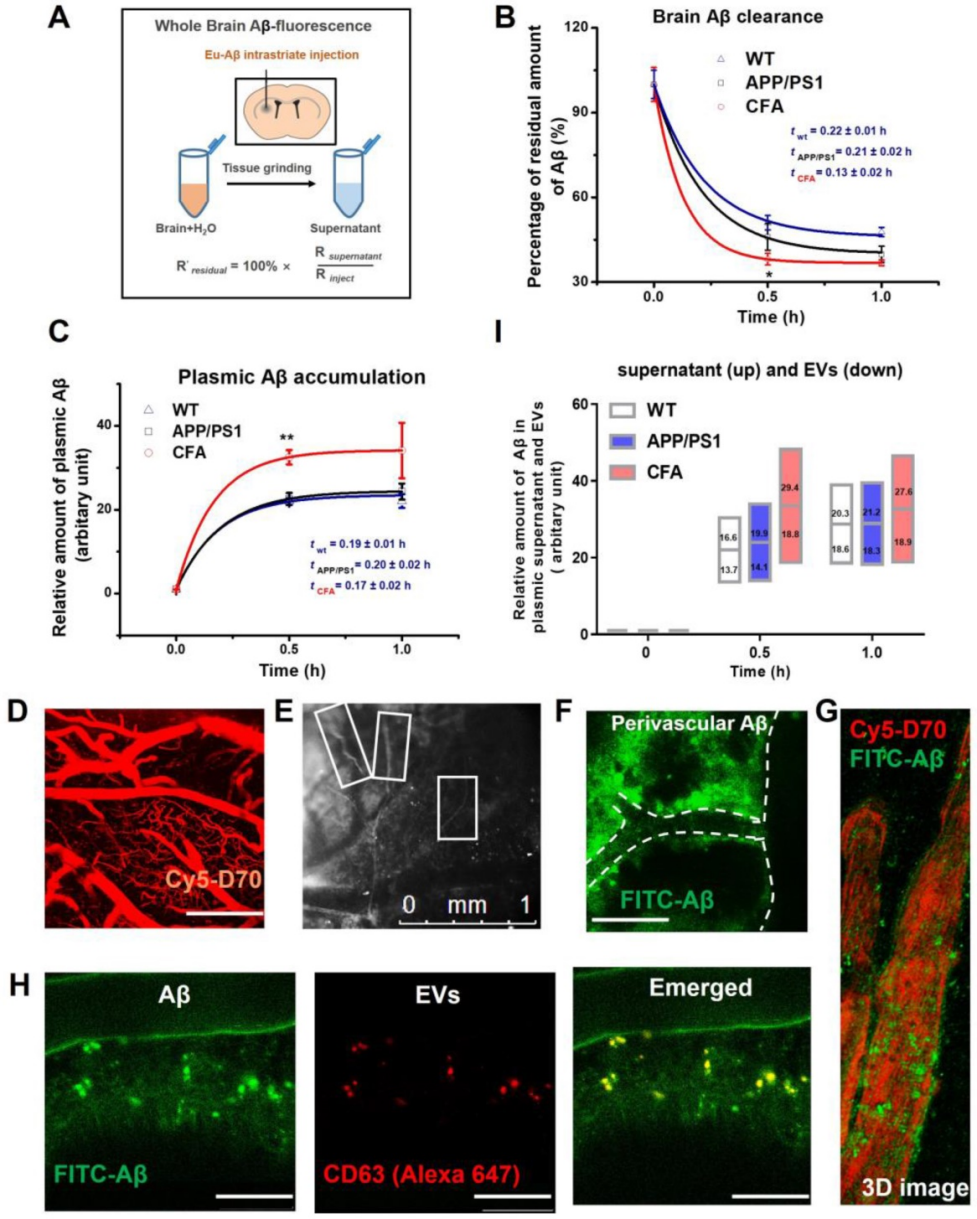
** CFA enhanced brain Aβ excretion in both EVs-bound and unbound forms in APP/PS1 AD mice**. Eu-labeled amyloid β (Eu-Aβ, 2µl) was injected into the mouse striatum and interstitial soluble amyloid Aβ clearing from the brain parenchyma was evaluated as described in Materials and Methods. (**A**) Schematic of Aβ intrastriatal injection and fluorescence measurement setup. (**B**) The time course of brain Aβ content (*P < 0.05, *n* = 4~6). (**C**) Time course of Aβ level in peripheral blood (**P < 0.01, *n* = 4~6). The fluorescence intensity of samples was presented as folds of plasma fluorescence blank. (**D**-**H**) *In vivo* imaging of Aβ flux in mouse cortex surface layer (0~240 μm) after intracisternal injection. **D**: cerebral vasculature visualized with intra-arterial Cy5-Dextran 70KD. Scale bars, 250 μm. < 0.5 h after intracisternal injection. **E**: capillaries surrounded by FITC-labeled Aβ highlighted by rectangle marks; **F**: FITC-labeled Aβ (Green) moving along the outside of cerebral surface blood vessels in a representative area (green circle) in **F**, Scale bars, 250 μm. **G**: 3D image of FITC-Aβ spots (Green) moving outside blood vessels. The fluorescence background of free FITC-Aβ was reduced manually to highlight the Aβ spots. (**H**) Co-localization of EVs and Aβ spots outside cerebral blood vessels. EVs was visualized with an Alexa 647-labeled CD63 antibody. Scale bars, 50 μm. (**I**) The relative amounts of free plasma Aβ and EVs-bound forms.

Nrf2 has a role in facilitating drug/metabolic waste excretion 21-24, 36, 37 . Therefore, we also tested whether CFA affected brain Aβ excretion *in vivo* in APP/PS1 transgenic mice, a well-recognized AD animal model 53. As shown in Fig. 7A-B, after intrastriatal injection 54 of Aβ_42_ labeled with a fluorescent lanthanide (i.e. Eu^3+^) complex, the brain content of fluorescent Aβ was observed to decrease rapidly with time. Data fitting to a model of one exponential decay (*y* = *Ae*^-*x*/*t*^ + *y*_0_) showed that the retention time constants (*t*) and maximal excretion of Aβ (*A*) in brain were 0.19±0.02 h and 53±1% for WT mice, 0.23±0.02 h and 61±1% for APP/PS1 mice, and 0.14±0.02 h and 64±2% for CFA(H)-treated APP/PS1 mice, respectively. Meanwhile, the levels of Aβ in peripheral blood were observed to increase reciprocally to the decrease of brain Aβ (Fig. 7C**)**. Data fitting to a model of one exponential growth (*y* = *y*_0_ + *A*(1 - *e*^-x/*t*^)) presented that the time constants (*t*) and relative amounts of plasma Aβ (*A*) were 0.19±0.02 h and 22.3±0.2 (WT mice), 0.20±0.02 h and 23.5±0.4 (untreated APP/PS1 mice), 0.17±0.02 h and 33.3±0.6 (CFA-treated AD mice, *P*<0.1 *vs* WT or APP/PS1), respectively. The results of decreased Aβ and increased plasma Aβ levels are in good agreement with each other. Overall, these results showed that CFA treatment significantly increased the rate and amount of brain Aβ clearance by 30~40% (*P*<0.01 *vs* WT or APP/PS1 mice).

To further investigate the potential pathways, we observed the Aβ flux in the brain parenchyma upon intracisternal injection of FITC-labeled Aβ using a two-photon laser scanning microscopy. A blood-brain barrier (BBB)-impermeable dye (Cy5-d70, intravenous injection) was used to visualize the cerebral vasculature (Fig. 7D). It was observed that labeled Aβ moved along the space outside the cortical surface blood vessel (Fig. 7E-G). It is noted that Aβ was viewed close to the vascular wall (Fig. 7G). Co-localization tests with intracisternal injection of both Alexa 647-labeled CD63 antibody (for staining the extracellular vesicles (EVs) and FITC-labeled Aβ (Figure 7H) revealed that the some Aβ particles overlapped very well with EVs, suggesting EVs as an Aβ-carrier in the process of Aβ excretion.

Next, the role of EVs in Aβ transport was investigated by measuring the distribution of Aβ between EVs and supernatant of plasma samples. The results (Fig. 7I) showed that Aβ is excreted from the brain in both EVs-bound and unbound forms. The ratio of unbound to EV-bound forms is about 54%:46% for WT control, 56%:44% for APP/PS1 mice, and 60%:40% for CFA-treated mice; and the ratios were not changed greatly with time. Therefore, about half of Aβ excretes from brain to blood in EV-bound forms. CFA treatment increased Aβ excretion in both pathways, albeit more enhancement for the unbound Aβ form. To our best knowledge, it is the first demonstration that EVs might participate in brain Aβ excretion and consistent with previous work showing that cellular Aβ release may be associated with exosomes 55.

### Coniferaldehyde preserves learning and memory function in APP/PS1 AD mice

**Figure 8:**
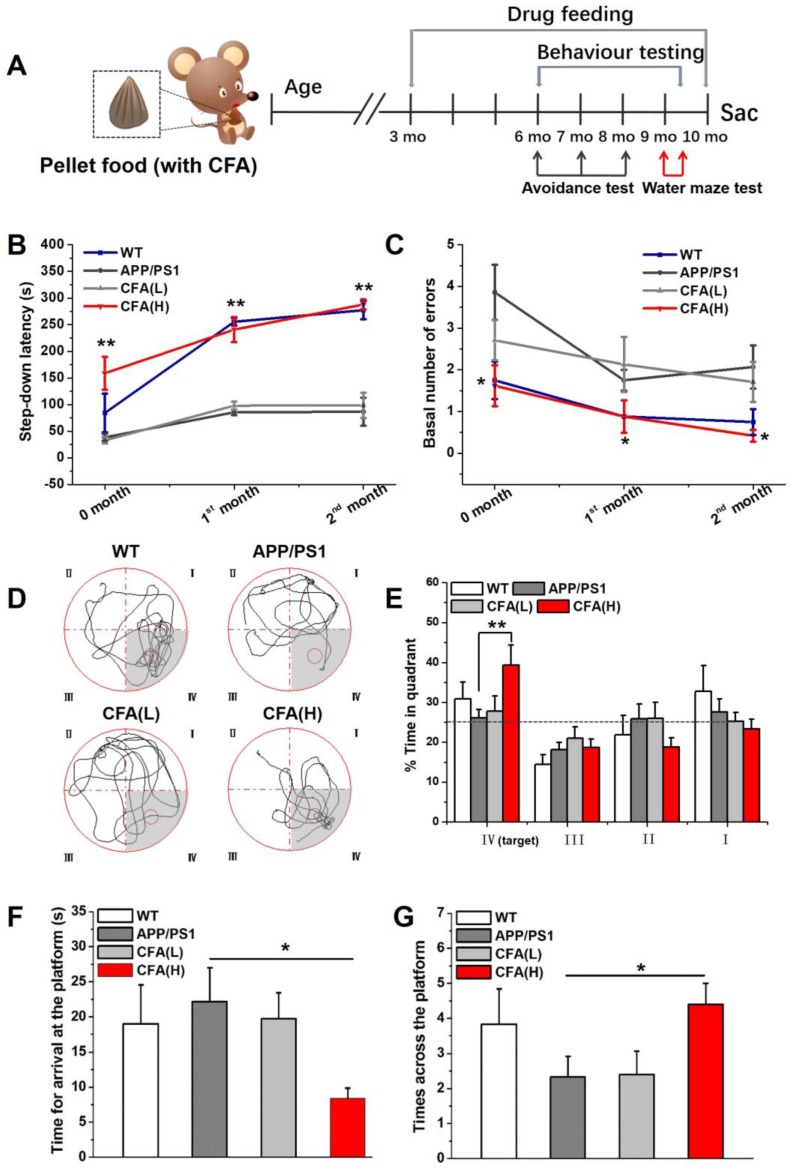
** Learning and memory protective effects of CFA in male APP/PS1 AD mice.** (**A**) Illustration of dosing and experimental design. CFA treatments on male APP/PS1 mice began in the 3^rd^ month by feeding the animals with pellet food containing desired amounts of CFA at a low dose (0.02 mmol kg^-1^day^-1^) and a high dose (0.2 mmol kg^-1^day^-1^) denoted CFA(L) and CFA(H), respectively. Step-down passive avoidance tests were conducted from 6 months of age to monitor the cognitive changes and Morris water maze tests were conducted at 9-10 months to evaluate spatial learning and memory. The littermate C57BL/6 mice (WT) were the negative control; (**B**) The step-down latency and basal number of errors (**C**) in the step-down passive avoidance tests. (**D**) Circular images display the representative swimming paths for the mice to locate the escape platform in the water maze during the 60 s test period. The small circle indicates the position of escape platform; (**E**) The percentage of time animals spent in the target quadrant (IV) compared to the other three quadrants (III, II, I); (**F**) Time to the first arrival in the escape platform position in the final water maze tests; (**G**) Times across the escape platform in the final water maze tests; Data represented as mean±SE (*n*=8~10). *P<0.05, **P<0.01 *versus* untreated APP/PS1 mice.

The ability of CFA to prevent AD-related phenotypes was tested in APP/PS1 transgenic mice. The male mice began receiving orally administered CFA at 3 months old (Fig. 8A), using two different dosing regimens, i.e. high dose, designated CFA(H) of 0.2 mmol kg^-1^d^-1^ and low dose, designated CFA(L) of 0.02 mmol kg^-1^d^-1^. Learning and memory capacity was measured with two different tests, starting after 3 months of CFA treatment (i.e. at 6 months of age).

In the step-down passive avoidance test, the first trial at 6 months old (Fig. 8B-C) showed that untreated APP/PS1 mice exhibited shorter step-down latency and greater learning error than wild type (WT) mice, suggesting decreased learning capacity; however, the 24 h memory for the electric shock was preserved in untreated APP/PS1 mice (Fig. S8). Retention tests at one and two months revealed that the step down latency was significantly higher, with the step down number lower, in WT and CFA(H) treated APP/PS1 mice compared to untreated or CFA(L) treated APP/PS1 mice, indicating that CFA treatment improved memory retention in APP/PS1 mice.

The learning and memory protection effect of CFA was further verified by the classical Morris water maze test. Theoretically, when an animal has completely lost its spatial memory, it will randomly swim, searching in the four quadrants for the hidden escape platform, while an animal that remembers the location of the platform will prefer a specific quadrant, as indicated by its staying time of >25% within that quadrant. As shown in Fig. 8D-E, untreated and CFA(L) treated mice showed no preference for the quadrant containing the platform, while WT mice spent the greatest time in the target quadrant. Remarkably, CFA(H) treated mice demonstrated significantly increased time in the target quadrant. Moreover, CFA(H) treated mice had a significantly reduced time to the first arrival at the location of platform (Fig. 8F), and significantly increased times across the platform (Fig. 8G) compared to untreated or CFA(L) treated APP/PS1 mice. The animal performance could be ranked in order of CA(H) > WT > (CA(L)≈AD control).

The ability of CFA (0.2 mmol kg^-1^d^-1^) to preserve learning and memory function of female APP/PS1 mice was also assessed (Fig. S9) and similar results were observed as in the male APP/PS1 mice. Overall, the experimental results indicated that APP/PS1 mice had impaired spatial memory compared to WT animals, but high CFA dosage treatment rescued the memory phenotype. Low dosage of CFA was overall ineffective despite improvement in certain aspects.

### Coniferaldehyde prevents neuronal loss in APP/PS1 AD mice

**Figure 9:**
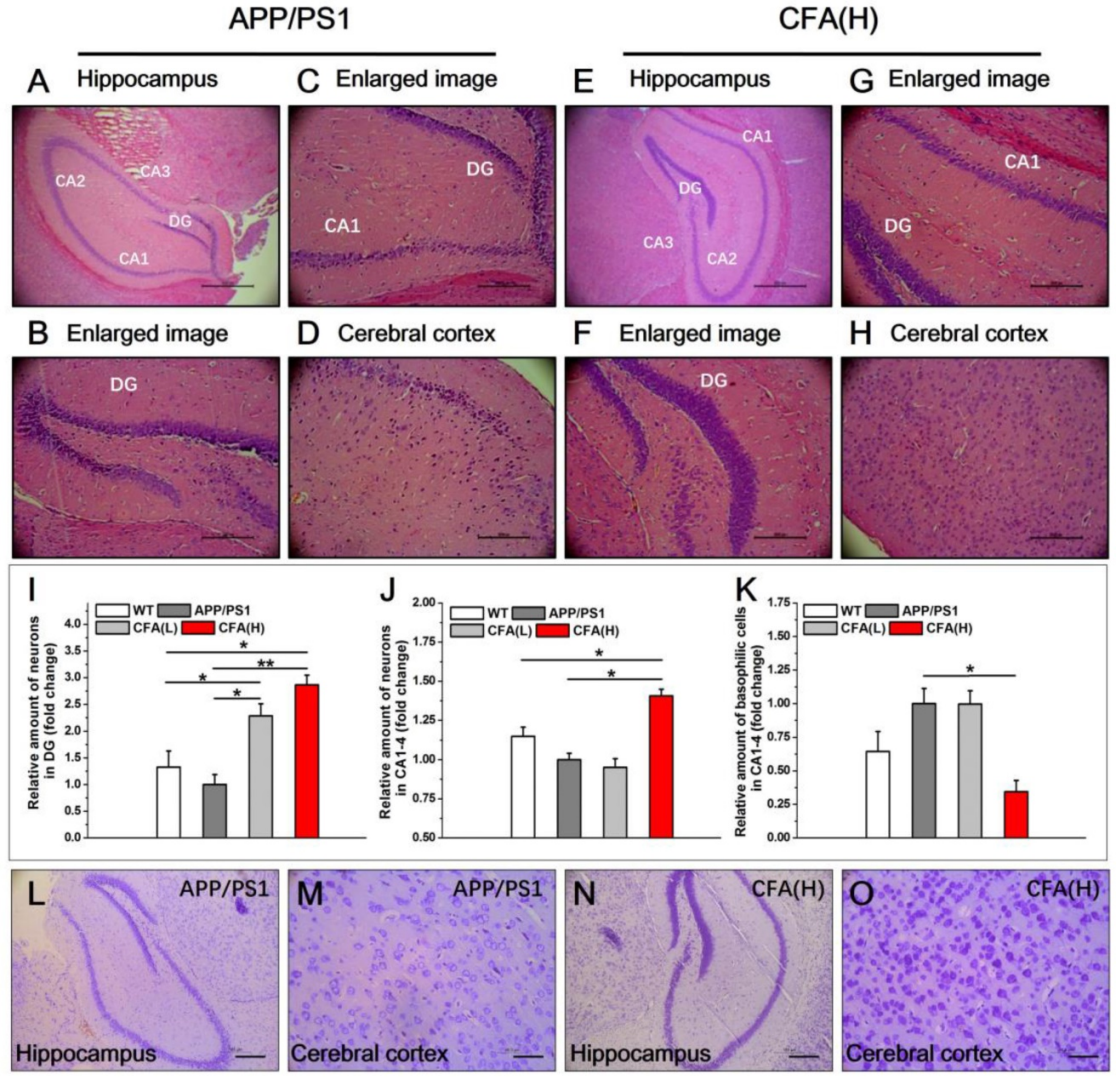
** Histopathological demonstration of protection effects of CFA in APP/PS1 AD mice.** (**A**-**H**) Representative images of hematoxylin-eosin (HE) staining of brain sections (hippocampus and cerebral cortex) of APP/PS1 AD model mice (male) upon CFA(H) treatment. Scale bars, 534.5 μm for 6.3× (**A**,**E**), 169.5 μm for 20× (**B**-**D**, **F**-**H**). Magnification: 6.3× (**A**, **E**), 20× (**B**-**D**, **F**-**H**). (**I**-**K**) Quantification of the total number of neurons in the DG (**i**), and the neurons (**J**) and basophilic cells (**K**) in CA1, CA2, CA3 and CA4. (**L**-**O**) Nissl staining of neurons in the brains APP/PS1 mice upon CFA treatment. Scale bars, 185 μm for (**L**, **N)**; 46.3 μm for (**M**, **O)**. The mice were 10 months old and had been treated with CFA in pellet food for 7 months. Results are mean±SE (*n*=6, with random selection). *P<0.05, **P<0.01, ***P<0.001*versus* untreated APP/PS1 or specific indication.

In untreated male APP/PS1 mice, consistent with previous reports 56, 57, substantial neuronal loss was apparent in the hippocampus (Fig. 9A-C) and in cerebral cortex (Fig. 9D), with many remaining neurons demonstrating basophilic staining and deeply stained and condensed nuclei. In contrast, no obvious pathological alterations were observed in CFA(H) treated mice (Fig. 9E-H). In addition, the dentate gyrus (DG) of CFA(H)-treated mice showed well preserved holonomic structure similar to WT mice (Fig. S10) and had obviously more cell layers than the untreated APP/PS1 control. When the number of surviving neurons was quantified, both high and low doses of CFA significantly increased the number of cells observed in the DG compared to WT and untreated APP/PS1 mice (Fig. 9I). In the hippocampus (CA1-CA4), CFA(H) significantly increased the number of surviving neurons (Fig. 9J) and decreased the number of basophilic neurons (Fig. 9K) compared to either WT or untreated APP/PS1 animals.

The results of Nissl staining analysis (Fig. 9L-O) revealed that CFA(H) treatment caused obviously more abundant and larger neuronal Nissl bodies in both hippocampus and cerebral cortex, indicating improvement of structure and functions of neuronal endoplasmic reticulum and golgi apparatus 58, 59 upon treatment with CFA(H).

### Coniferaldehyde prevents toxic Aβ accumulation in APP/PS1 AD mice

**Figure 10:**
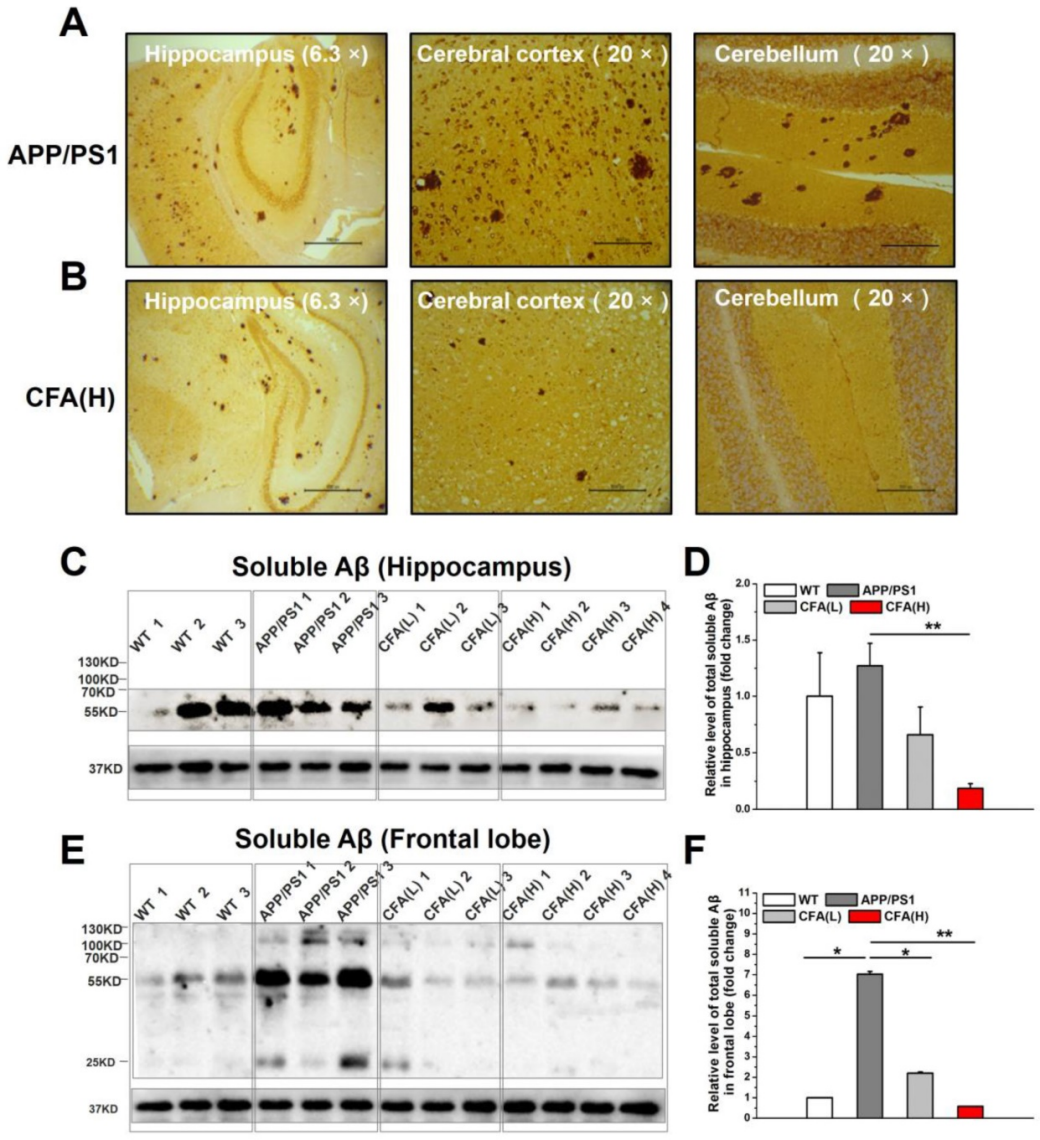
** CFA treatment eliminated brain Aβ deposition and toxic Aβ accumulation**. (**A-B**) Representative images of brain Aβ deposition. The brain samples (hippocampus, cerebral cortex and cerebellum) were immunostained with the Aβ antibody (6E10) in untreated APP/PS1 control (**A**) and CFA(H)-treated mice (**B**). Scale bars, 534.5 μm for 6.3×, 169.5 μm for 20×. (**C-F**) Aβ oligomerization in APP/PS1 mice upon CFA treatment. Western blot analysis and quantification of soluble Aβ peptides in hippocampal lysates (**C, D**) and frontal lobe lysates (**E, F**). The mice were 10 months old and had been treated with CFA in pellet food for 7 months. Results are mean±SE (*n*=6, with random selection). *P<0.05, **P<0.01, ***P<0.001*versus* untreated APP/PS1 or specific indication.

Aβ plaques are one important sign in AD pathology 4, 15, 16 recapitulated in the APP/PS1 model. Histological analysis of Aβ peptides revealed significant Aβ plaques in hippocampus, cerebral cortex and cerebellum, and intracellular Aβ staining (Fig. 10A) in untreated APP/PS1, but not WT mice. In comparison, in CFA(H)-treated mice Aβ plaques were significantly reduced in hippocampus and cerebral cortex, and absent in cerebellum (Fig. 10B).

Because the soluble Aβ oligomers (also known as Aβ-derived diffusible ligands, ADDLs) are suggested as more neurotoxic species 12, 20, 60, 61 than Aβ deposits, the levels of soluble Aβ oligomers upon CFA treatment were determined. The results showed that the amounts of soluble Aβ oligomers in CFA-treated groups were drastically reduced in both hippocampus (Fig. 10C-D) and frontal cortex (Fig. 10E-F) in a dose-dependent manner. Notably, the level of soluble Aβ oligomers in hippocampus of WT mice was elevated despite the lack of Aβ plaques; this may have indicated an aging-related decrease of brain metabolite clearance even in the healthy animals.

Taken together, the results above demonstrate that CFA treatment effectively promotes brain Aβ clearance and thus attenuates both soluble toxic Aβ levels (Fig. 10C-F) and subsequent insoluble Aβ deposition (Fig. 10B and S11).

## Discussion

The present work aimed to discover safe agents that promote neural cell viability, protect neurons under mitochondrial stress, and can promote Aβ clearance for early stage AD treatment. Considering the role of Nrf2 in regulating major cellular defense systems 21-24, we developed a strategy to screen a series of Nrf2 agonists *in vitro* and revealed promising properties of CFA. Intriguingly, CFA demonstrated significantly improved the cell viability both with and without Aβ stress or other mitochondrial toxins (MPP^+^ and rotenone) (Fig. 1). As expected, CFA strongly activated Nrf2 in neuronal cells both *in vitro* (Fig. 2) and *in vivo* (Fig. 6) and activation of Nrf2 would account for most of the actions of CFA (Fig. 2).

We demonstrate the importance of Nrf2 activation in the neuroprotective effects observed, and hypothesize that its downstream effects are key to the multi-modal, widespread ameliorating effects of CFA on Aβ-induced pathophysiology. Nrf2 is a master transcriptional regulator mediating expression of a variety of antioxidant enzymes and phase II+ enzymes and transporters 21-24, 36, 37. Under the resting state, Nrf2 is sequestered by Keap1 and targeted for rapid ubiquitin-mediated degradation 21-24, 36, 37. Upon oxidative stress, Nrf2 is released from Keap1 and phosphorylated, allowing Nrf2 to translocate to the nucleus and start downstream gene expression. Therefore, Nrf2 activation would include signs such as increase of Nrf2 level, nuclear translocation, and expression of downstream genes (e.g. hemeoxygenase 1, HO-1) 21-24, 36-38, all of which were demonstrated in our study upon treatment with CFA. Structurally related molecules, e.g., those containing the α,β-unsaturated carbonyl group, have previously been shown to activate Nrf2 by covalently linking to Keap1, thereby releasing its normal constitutive inhibition of Nrf2 62, 63, and leading to autoinduction of Nrf2 by its binding to its own promoter. Given the widespread effects of Nrf2 and its downstream targets, it is not surprising that CFA, as an Nrf2 activator, leads to parallel alterations in multiple pathways and cellular functions that are essential in AD pathogenesis 64-66 as demonstrated here. However, the full range of Nrf2 effects, as well as their mechanisms of action mediated by its targets, are an area of active study, and not fully elucidated. Thus, while questions remain about which Nrf2 targets link each of the observed functional improvements, it is quite plausible that this important regulator is a key step in their initiation.

Protection of mitochondrial structure and function is emphasized here because: (i) mitochondria are crucial for cell viability and (ii) are one major target of AD pathogenesis 18, 19, 41. In fact, bioenergetic deficit is an early aspect of AD pathology closely related to the human disease 18, 19, 41. Underlying the energy deficiency would be the disruption of the balance of mitochondrial fission and fusion, which leads to mitochondrial fragmentation and reduced mitochondrial density 42-44, and the impairment of mitochondrial oxidative phosphorylation, which causes a shift of the metabolic pattern towards the primitive fermentation of glucose (i.e. glycolysis, as marked by elevation of PKM2) that is similar to the Warburg effect observed in cancer cells 45, 46. As expected, CFA treatment effectively corrected the morphological and functional alterations in mitochondria induced by Aβ stress (Fig. 3). Further investigation revealed that CFA significantly reduced the level of Drp1 *in vitro* (Fig. 4) and membrane-associated Drp1 *in vivo* (Fig. S5). These results are consistent with previous studies of Drp1 on mitochondrial fragmentation induced by several factors including Aβ stress 48, 49, 67 and suggest that the recovery of mitochondrial morphology likely involves reduced expression or recruitment of Drp1 to the membrane. CFA treatment also enhanced mitochondrial oxidative phosphorylation capacity (Fig. 3) while reducing Aβ-stimulated glycolysis, including normalization of PKM2 expression (Fig. 4), thus restoring the cellular bioenergetics. However, the detailed mechanisms by which CFA treatment and subsequent Nrf2 activation accomplish these effects remain to be elucidated. A number of pathways by which Nrf2 alters ATP production have been reported, but the effects are dependent on the mechanism of activation, functional context, and cell type 65, 68. Nonetheless, Nrf2 activation was shown to up-regulate mitochondrial biogenesis genes with effects on improving the efficiency of oxidative phosphorylation and ultimately synthesis of ATP 39, 65, while Nrf2 deficiency would incline mitochondrial energy metabolism toward glycolysis 66. Hence, while we herein speculated a key role of Nrf2 activation for mitochondrial protection by CFA, its details require further detailed study.

Likely as a result of correcting the structural and functional alteration of mitochondria 69, 70, CFA treatment of SH-SY5Y cells increased the activation of Akt/PKB. Consequently, CFA caused phosphorylation-dependent inactivation of Tau kinase GSK-3β and decrease of p-Tau (S262 and S422) (Fig. 5). These results suggested that CFA is potentially beneficial in reducing tauopathy, though this aspect is waiting for further investigation.

Considering the role of Nrf2 in metabolism and excretion of drugs/wastes/toxins, the effect of CFA on Aβ clearance was tested using an *in vivo* assay (Fig. 7). The results showed that Aβ species were transported from brain to blood in free and EV-bound forms, and CFA treatment significantly improved both the rate and capacity of Aβ excretion (Fig. 7). The detailed pathways for enhanced Aβ excretion by CFA need to be investigated further. Previous works 71, 72 have proposed several mechanisms: blood brain barrier (BBB)-specific efflux, interstitial fluid (ISF) bulk flow and cerebrospinal fluid absorption. Early studies regard BBB as the major pathway for brain Aβ excretion 72. As Nrf2 up-regulates the endothelial P-glycoprotein (P-gp) 39, CFA might also promote Aβ efflux transport *via* P-gp on BBB. Alternatively, Nedergaard and colleagues recently suggested that ISF bulk flow facilitated by astroglial aquaporin-4 (AQP4) channels (known as the glymphatic system) may contribute to a larger portion of extracellular Aβ clearance 73-77. As we observed that: (i) about half amount of Aβ species were transported out in EV-bound form and (ii) the Aβ flux moved along the outside space of cortical blood vessels (Fig. 7E-G), the proposed glymphatic drain path 54, 78. Nonetheless, we expect that clearance of Aβ, whether *via* the BBB or the glymphatic pathway, to have a major role in the action of CFA.

Together, all the results above prompted us to examine the preventive and protective effects of CFA on AD pathology *in vivo* on the APP/PS1 transgenic mouse, a well-recognized AD animal model 53, 79. Although APP/PS1 transgenic mice do not recapitulate all aspects of AD pathology, they do undergo amyloid pathogenesis. Observable amyloid deposition begins at the age of 4~5 months and behavioral deficits after 6 months 80, 81. Importantly, certain crucial neuronal functions, i.e., inactive Nrf2, are quite similar to that of AD patients (Fig. 6). In addition, other important aspects of AD pathology such as Tau hyperphosphorylation normally appear in the later stages following Aβ overproduction 50, 51. Thus, the APP/PS1 transgenic mouse model is quite suitable for observing the effects of AD preventive drugs on the elevation of amyloid and its consequences, whether as initiating events or key elements facilitating AD progress in the early stages of AD 4, 18, 19, 41.

We started dosing animals with CFA at 3 months (Fig. 8A) when amyloid deposits are not observable, but the mice already exhibit early pathological changes, i.e. swollen cholinergic axons and cholinesterase positive reactions in the cortex and hippocampus 79, 82-84. The results were of note: (i) CFA at the effective dosage (0.2 mmol kg^-1^day^-1^) effectively preserved learning and memory function of the model mice (Fig. 8). (ii) CFA treatment significant reduced AD-like pathological amyloid features (Fig. 9 and 10), i.e. well-preserved DG holonomic structure, improved number of surviving neurons, decreased the number of basophilic cells and improved neuronal endoplasmic reticulum and golgi apparatus, etc.; (iii) CFA treatment almost cleaned the Aβ deposits in major brain regions (Fig. 10 and S11) and essentially abolished the toxic soluble Aβ species (Fig. 10), which conceivably could account for the reduction of neuronal lesions and improved cognitive function.

Another noteworthy effect of CFA is the recovery of Nrf2 activity *in vivo* (Fig. 6). Previously, Nrf2 was found inactive in AD brain despite the presence of oxidative stress 25, 26. Conceivably, several Nrf2-dependent antioxidant enzymes such as SOD1 and catalase were also found reduced in human AD brains 26, 85. In APP/PS1 mice, overexpression of Nrf2 protected neurons against Aβ toxicity and recovered spatial learning 22, 26, 66 but knockout of Nrf2 aggravated oxidative damage 86. Herein, we found that Nrf2 was down-regulated Aβ-expressing neural cells (Fig. S3), while in APP/PS1 mice, Nrf2 was both down-regulated and inactive (Fig. 6). Moreover, brain Aβ excretion in APP/PS1 mice showed a reduced rate (Fig. 7). Hence, we expect a major role of Nrf2 activation in the action of CFA despite lack of elucidation of the detailed pathways in the future works. Nevertheless, the present work supports Nrf2 as an enticing target for AD therapeutic agent discovery.

The preventive/protective effects of CFA *in vitro* and animal models suggest further studies of CFA in human subjects, especially in patients already diagnosed with cognitive impairment. An important benefit of CFA as a potential AD treatment is that it is a legal food flavoring (EFSA FL-No. 05.155), essential in wine brandy/whiskey 29, 87. CFA is also an active ingredient in many herbals, such as propolis and bamboo shavings. Analysis of brain CFA concentrations in mice upon oral administration (0.2 mmol/kg) revealed a quick absorption of CFA with a peak at about 1h (Fig. S12) and the data may fit into a compartmental pharmacokinetic model, suggesting that CFA can easily pass through BBB. More importantly, CFA shows a safety profile (Oral LD50: mouse, 300 mg/kg; rabbit 3200 mg/kg; rat, 980 mg/kg) well within the effective range tested here. This factor is especially appealing for a potential preventative medication, as administration to patients not currently exhibiting severe disease particularly requires high safety and low side effects.

In summary, the current investigation demonstrates that a food flavoring CFA, when given during early stages of AD-related processes, could greatly diminish amyloid pathogenesis in APP/PS1 transgenic mice. The mechanism of the actions of CFA involved protective actions probably include the protection of mitochondrial structure, dynamics, and energy metabolism from Aβ toxicity particularly as well as enhancement of Aβ excretion. The enhanced Aβ secretion is likely *via* the glymphatic and/or BBB efflux systems in both free and exosomes and microvesicles (EVs)-bound forms, emphasizing the need of further investigation on EVs in Aβ transportation. Furthermore, our results overall support Nrf2 activation as an essential mechanism for the therapeutic effects of CFA. With good safety and pharmacokinetic profiles, CFA would be an ideal candidate for further clinical tests as a preventive medicine of AD.

## Supplementary Material

Supplementary methods and figures.

## Abbreviations

ADDLs: Aβ-derived diffusible ligands
APP: amyloid precursor protein
APP/PS1: APPswe/PS1dE9
ATRA: all-trans-retinoic acid
CFA: coniferaldehyde
DG: dentate gyrus
MPP^+^: 1-methyl-4-phenylpyridinium
Nrf2: NF-E2-related factor 2
MWM: Morris water maze
Rot: Rotenone
WT: wild type
