# Supplementary material for: Coniferaldehyde attenuates Alzheimer's pathology via activation of Nrf2 and its targets
Source: Theranostics. 2020 Jan 1;10(1):179–200. doi: 10.7150/thno.36722 (PMC6929631; doi:10.7150/thno.36722)
Supplement: Supplementary file 1 — Supplementary methods and figures. [file thnov10p0179s1.pdf]

Coniferaldehyde attenuates Alzheimer's pathology *via* activation of Nrf2 and its targets

Yaqiong Dong[1], Tessandra Stewart[2], Lidan Bai[1], Xue Li[1], Ting Xu[1], Jeffrey Iliff[3], Min Shi[2], Danfeng Zheng[4], Lan Yuan[1], Taotao Wei[5], Xiaoda Yang[1]✉, and Jing Zhang[2,4]✉

Supplemental Information

Supplemental Methods

Nuclear Extraction

The SH-SY5Y cells were grown on 6-well culture dishes. After treatment with 100 μM CFA for 36 h at 37 °C, nuclear–cytoplasmic fractionation was conducted using the Nuclear and Cytoplasmic Extraction Reagents kit (Beyotime Biotech, China) according to the manufacturer's protocol. The Nrf2 expression of nuclear extracts was analyzed by western blot and quantified.

Measurement of intracellular ROS

The SH-SY5Y cells were grown on 6-well culture dishes. After CFA treatment, the cells were collected and incubated with 10 μM of DCFH-DA at 37°C for 20 min. Then cells were washed three times with FBS-free DMEM medium. The fluorescence intensity of DCF was determined using a flow cytometer (Beckman Coulter, USA) with the excitation/emission wavelength at 488/525 nm.

Assessment of mitochondrial membrane potential

Fluorescent probe JC-1 (Bridgen, China) was used to detect the mitochondrial

membrane potential ($\Delta\Psi_m$) of the SH-SY5Y cells upon drug treatment as described. Briefly, the SH-SY5Y cells were grown on 6-well culture dishes. After CFA treatment, the medium was removed and fresh medium containing JC-1 (1×) was added and incubated for 20 min at 37 °C. Then the cells were washed twice with JC-1 staining buffer (1×) and observed on a fluorescence microscope (Olympus, Japan) with excitation/emission at 550/600 nm (the red fluorescence channel) or excitation/emission 485/535 nm (green fluorescence channel).

Pharmacokinetics in brain

To determine the kinetics of CFA in brain, ICR mice (n=5~6) were treated with CFA (0.2 mmol/kg) by gavage. Then, 5min, 0.5 h, 1 h, 2 h, 3 h, 4 h, and 6 h after the administration, the brains of mice were harvested and added to 5 times the mass of pre-chilled deionized water and homogenized with a bullet blender (Gene Company Limited, Hong Kong). Then homogenates were centrifuged at 5,000×g for 15 min and the supernatant was then collected. Meanwhile, sterile 0.9 % saline was also intravenously injected into mice as control (0 h). As fluorometry is more specific than absorptiometry, it is frequently used to determine a biogenic substance present in a complex mixture. So, the concentration of CFA was examined according to the previous fluorometric method [1]. The reagent for the fluorescence reaction was freshly prepared by mixing 1 ml of 120 mM methanolic Isonicotinylhydrazine (INH), 1 ml of 60 mM methanolic $AlCl_3$, and 1 ml of MeOH. The reagent (3 ml) was added to 1 ml of the CFA sample solution in a test tube. The mixture was then warmed at 50 °C for 10 min. After cooling in running H_2O for 3 min, the fluorescence intensity was immediately measured.

A calibration curve was prepared using different concentrations of CFA ranging from 5 to 100 μmol/L by the same procedure as above. The fluorescence intensity was then quantified by fluorescence spectrophotometer (Cary Eclipse, USA) with excitation and emission wavelengths at 400 nm and 502 nm, respectively, against a blank.

Supplemental Figure Legends

Figure S1. The effects of coniferaldehyde (CFA) on the expression of BDNF in SH-SY5Y cells.

Figure S2. CFA treatment increased Nrf2 expression of nucleus in SH-SY5Y cells.

Figure S3. CFA treatment increased Nrf2 and HO-1 expression in SH-SY5Y cells.

Figure S4. Nrf2 siRNA pretreatment for 24 h effectively decreased Nrf2 expression in SH-SY5Y cells.

Figure S5. Effects of coniferaldehyde (CFA) on membrane-associated Drp1 levels in brain.

Figure S6. Effects of coniferaldehyde (CFA) on mitochondrial membrane potential ($\Delta\Psi_m$).

Figure S7. Effects of coniferaldehyde (CFA) on ROS of SH-SY5Y cells (neo, APPwt and APPswe).

Figure S8. The step-down latency (A) and basal number of errors (B) in 24 h retention tests in the step-down passive avoidance tests of WT mice and APP/PS1 mice with/without coniferaldehyde (CFA) treatment.

Figure S9. Step-down passive avoidance tests were conducted from 6 months of age to

monitor the cognitive changes of female APP/PS1 mice.

Figure S10. Representative images of hematoxylin-eosin (HE) staining of brain sections (hippocampus, cerebral cortex and cerebellum) of WT mice and APP/PS1 mice with/without coniferaldehyde (CFA) treatment.

Figure S11. Insoluble Aβ petides in WT mice and APP/PS1 mice with/without coniferaldehyde (CFA) treatment.

Figure S12. The concentration time curve of coniferaldehyde (CFA) in mice brain upon gavage administration.

Supplemental Figures

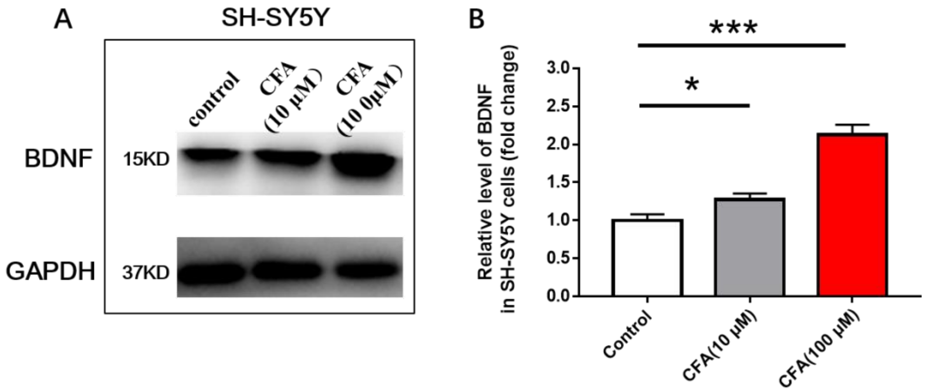

Figure S1. The effects of coniferaldehyde (CFA) on the expression of BDNF in SH-SY5Y cells. SH-SY5Y cells were treated with CFA for 36 h before quantification of cell viability by MTS assay. The assays were conducted as described in Materials and Methods. Results are mean±SD (n=3). **P*<0.05, ****P*<0.001.

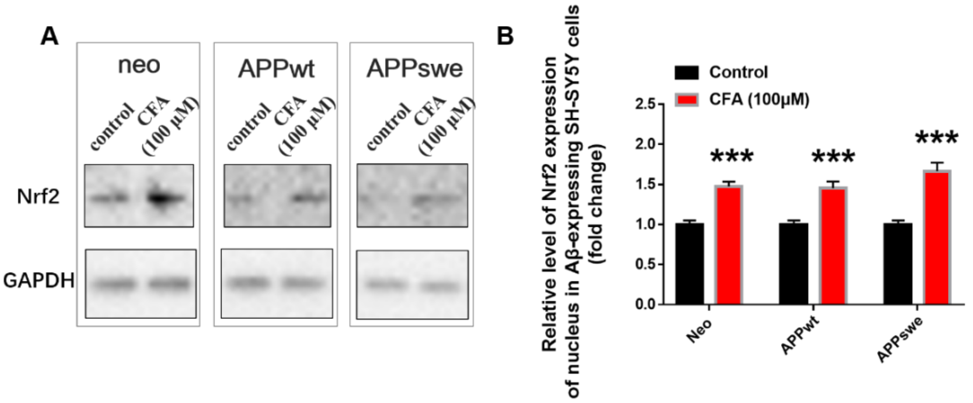

Figure S2. CFA treatment increased nuclear Nrf2 expression in SH-SY5Y cells. SH-SY5Y cells were treated with CFA for 36 h. Nuclear extracts were obtained, and Nrf2 expression was analyzed by western blot (A) and quantified (B). Results are mean±SD (n=3). **P*<0.05, ****P*<0.001.

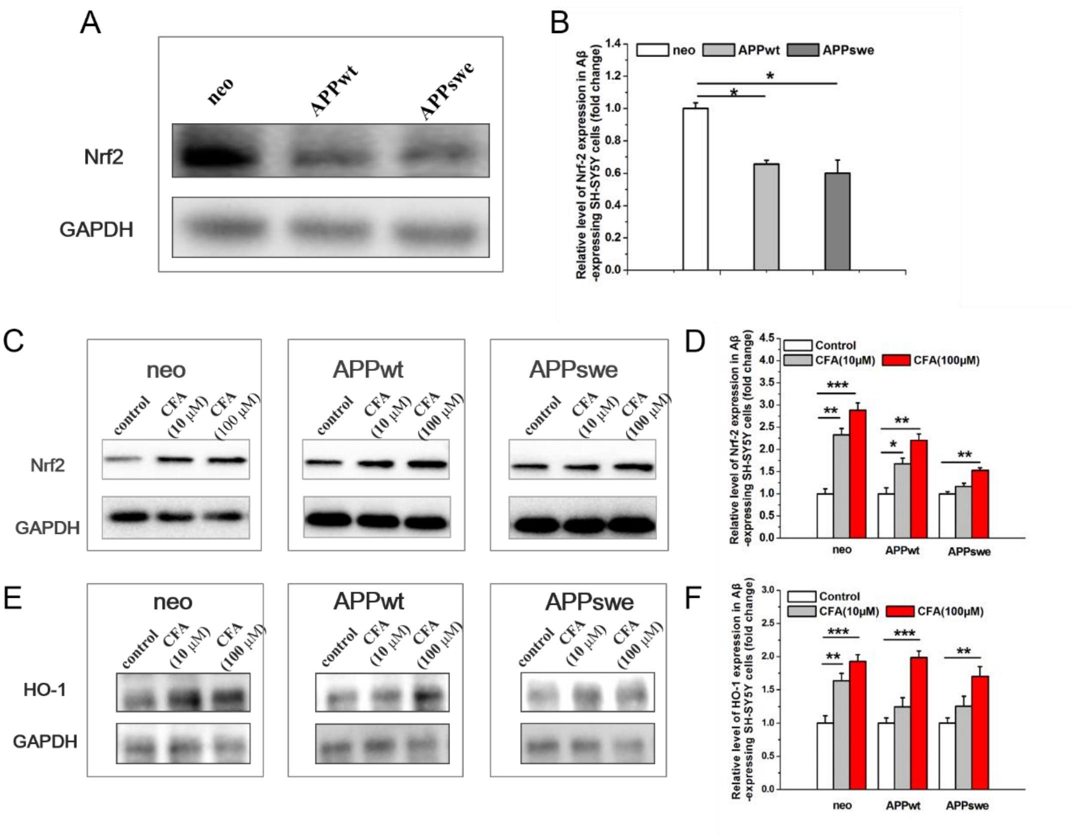

Figure S3. CFA treatment increased Nrf2 and HO-1 expression in SH-SY5Y cells. (**A, B)** The basal Nrf2 levels of SH-SY5Y cells (neo, APPwt and APPswe). Nrf2 expression was analyzed by western blot (**A**) and quantified (**B**). (**C-F**) Nrf2 and HO-1 expression was analyzed by western blot (**C, E**) and quantified (**D, F**). Nrf2 and HO-1 expression were normalized to GAPDH expression and the fold changes over their own control are shown. The assays were conducted as described in Materials and Methods. Results are mean±SD (n=3). *P<0.05, **P<0.01, ***P<0.001.

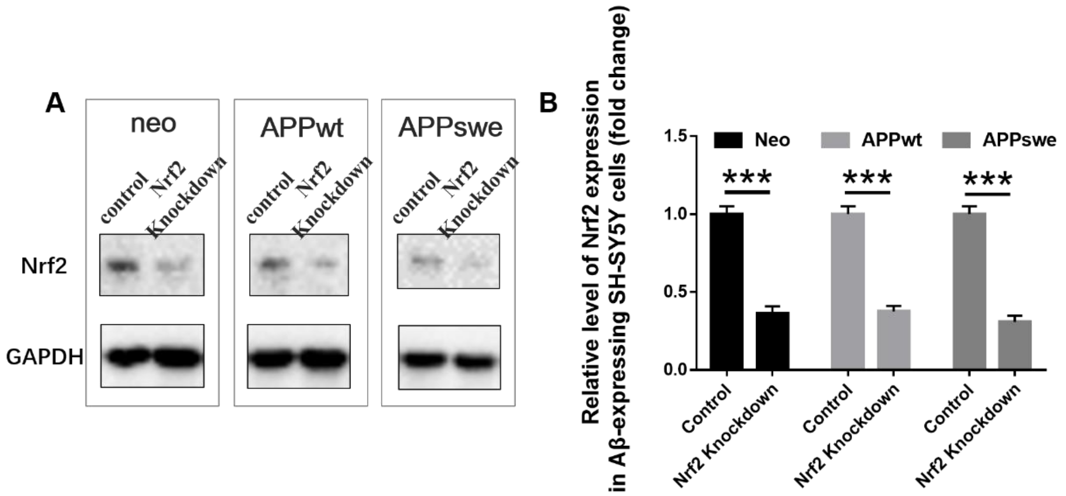

Figure S4. Nrf2 siRNA pretreatment for 24 h effectively decreased Nrf2 expression in SH-SY5Y cells. Nrf2 expression was analyzed by western blot (**A**) and quantified (**B**). The assays were conducted as described in Materials and Methods. Results are mean±SD (n=3). ***P<0.001.

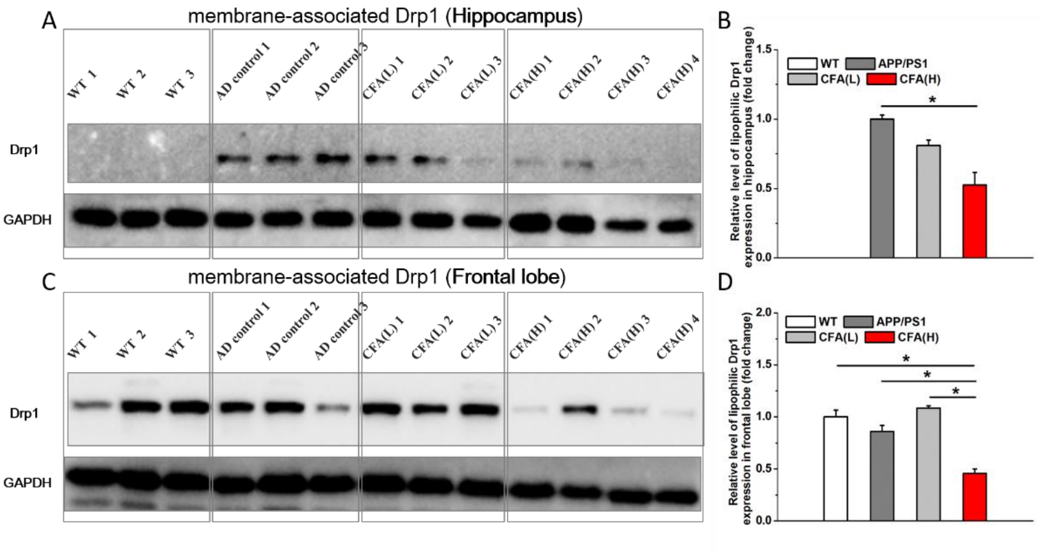

Figure S5. Effects of coniferaldehyde (CFA) on Membrane-associated Drp1 levels in brain. Western blot analysis of lipophilic Drp1 levels in hippocampus (**A**,**B**) and frontal cortex (**C**,**D**) of WT mice and APP/PS1 mice brains with/without CFA treatment. The assays were conducted as described in Materials and Methods. Results are mean±SE (n=6, with random selection). *P<0.05.

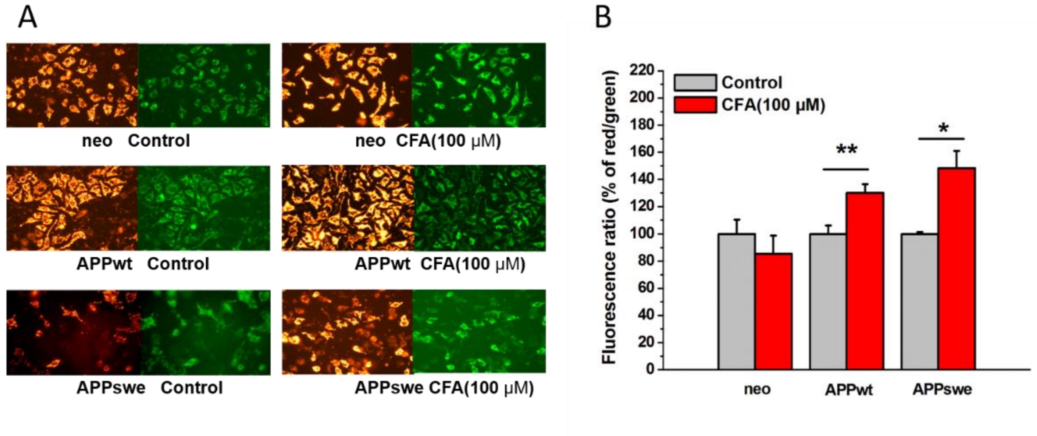

Figure S6. Effects of coniferaldehyde (CFA) on mitochondrial membrane potential ($\Delta\Psi_m$). (**A**,**B**) The change of $\Delta\Psi_m$ was expressed in SH-SY5Y cells (neo, APPwt and APPswe) by alteration of fluorescence ratio (red/green) treated with or without CFA (100 μM). The JC-1 dye (Bridgen, China) was used to detect the mitochondrial membrane potential ($\Delta\Psi_m$). Briefly, ffter CFA treatment, the cells were incubated with fresh medium containing JC-1 (1×) for 20 min at 37 °C. After twice wash, the cells were observed on a fluorescence microscope (Olympus, Japan) with excitation/emission at 550/600 nm (the red fluorescence channel) or excitation/emission 485/535 nm (green fluorescence channel). Results are mean±SD (n=3). *P<0.05, **P<0.01.

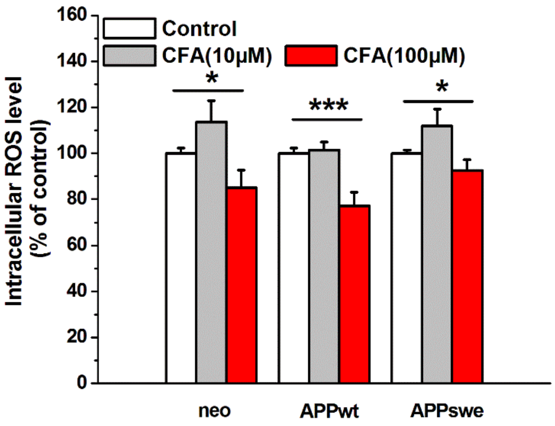

Figure S7. Effects of coniferaldehyde (CFA) on ROS of SH-SY5Y cells (neo, APPwt and

APPswe). After CFA treatment, the SH-SY5Y cells were collected and incubated with 10 μM of DCFH-DA at 37°C for 20 min. Then cells were washed three times with FBS-free DMEM medium. The fluorescence intensity of DCF was determined using a flow cytometer (Beckman Coulter, USA) with the excitation/emission wavelength at 488/525 nm. Results are mean±SD (n=3). **P*<0.05, ****P*<0.001.

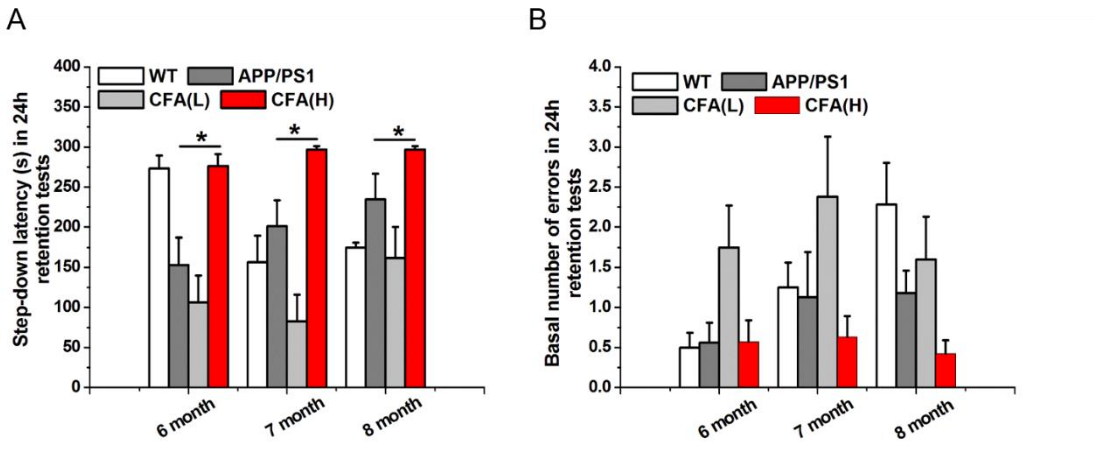

Figure S8. The step-down latency (A) and basal number of errors (B) in 24 h retention tests in the step-down passive avoidance tests of WT mice and APP/PS1 mice with/without coniferaldehyde (CFA) treatment. The tests were conducted as described in Materials and Methods. Results are mean±SE (n=8-10). **P*<0.05.

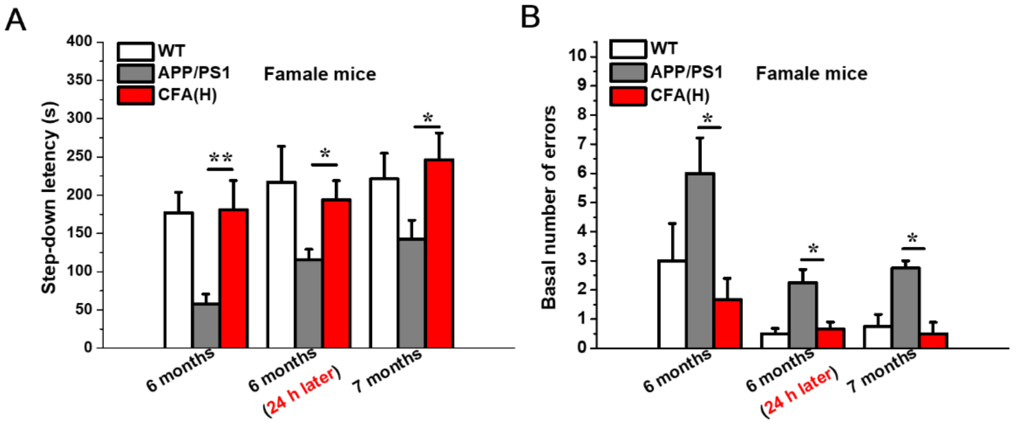

Figure S9. Step-down passive avoidance tests were conducted from 6 months of age to monitor the cognitive changes of female APP/PS1 mice. The step-down latency (**A**) and basal number of errors (**B**) in the step-down passive avoidance tests of female WT mice and APP/PS1 mice with/without coniferaldehyde (CFA) treatment. The tests were conducted as described in Materials and Methods. Results are mean±SE (n=5). **P*<0.05, ***P*<0.01 *versus* untreated APP/PS1 control.

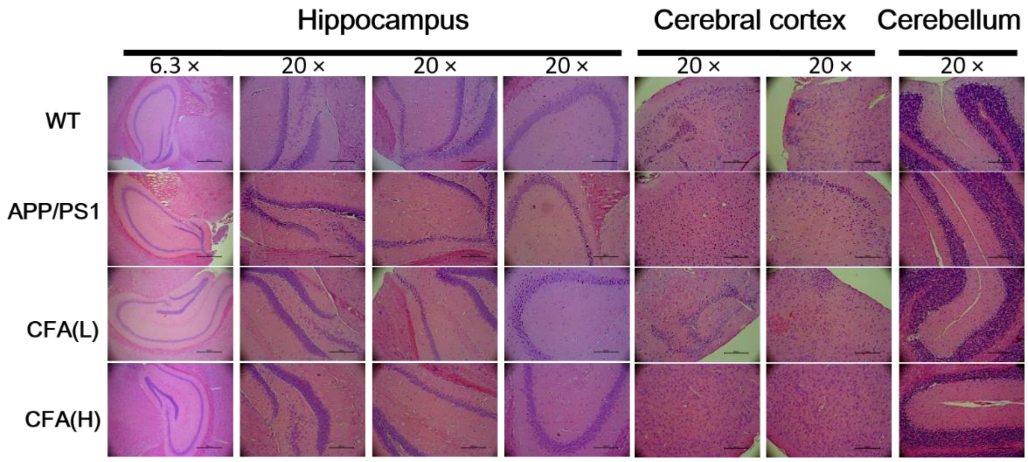

Figure S10. Representative images of hematoxylin-eosin (HE) staining of brain sections (hippocampus, cerebral cortex and cerebellum) of WT mice and APP/PS1 mice with/without coniferaldehyde (CFA) treatment. The assays were conducted as described in Materials and Methods. Scale bars, 534.5 μm for 6.3×, 169.5 μm for 20×.

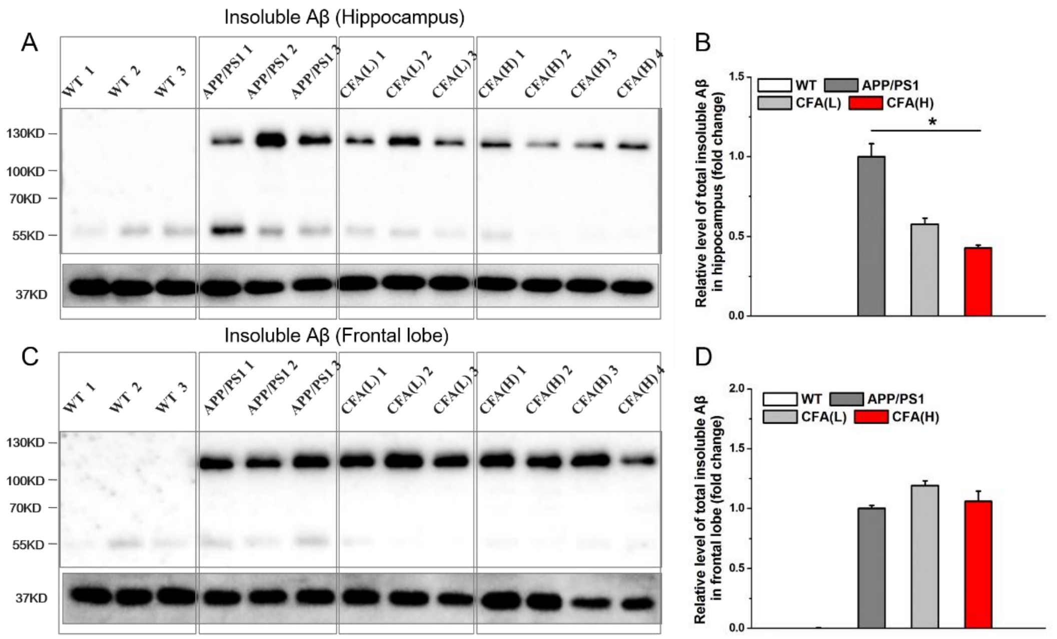

Figure S11. Insoluble Aβ petides in WT mice and APP/PS1 mice with/without coniferaldehyde (CFA) treatment. Western analysis and quantification of insoluble Aβ peptides in hippocampus lysates (**A**,**B**) and frontal lobe lysates (**C**,**D**). The assays were conducted as described in Materials and Methods. Results are mean±SE (*n*=6, with random selection). **P*<0.05.

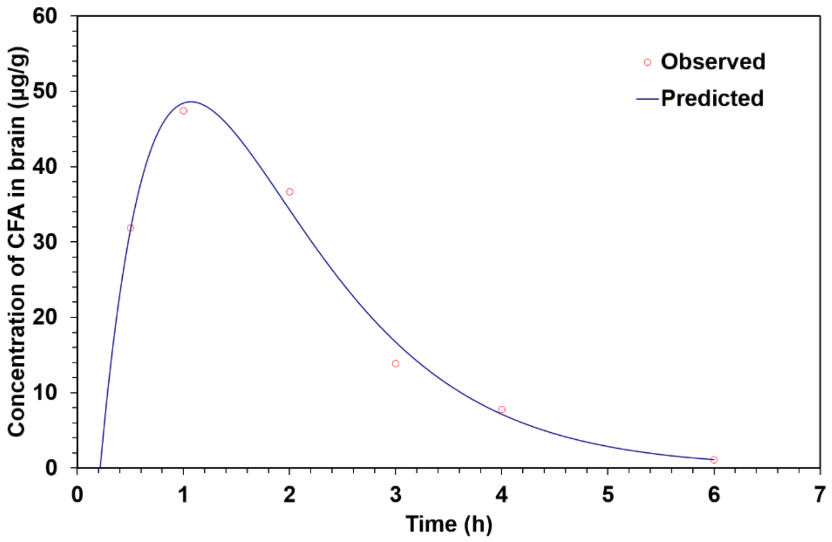

Figure S12. The concentration time curve of coniferaldehyde (CFA) in mice brain upon gavage

administration. ICR mice (*n*=6) were treated with CFA by gavage in 0.2 mmol/kg. At 5min, 0.5 h, 1 h, 2 h, 3 h, 4 h, and 6 h, the brains of mice were harvested. Pre-chilled deionized water was added to the samples (5 times by mass), followed by homogenization with a bullet blender (Gene Company Limited, Hong Kong). Then homogenates were centrifuged at 5,000×g for 15 min and the supernatant was collected. The concentrations of CFA were determined using a fluorescence spectrometric method [1]. Briefly, the diluted samples (1.0 mL) were mixed with 1 mL of 120 mM Isonicotinylhydrazine (INH) and 1 mL of 60 mM $AlCl_3$ in methane solution. The mixture was then left at 50 °C for 10 min to complete fluorescence reaction. Finally the fluorescence intensity was measured on a fluorescence spectrophotometer (Cary Eclipse, USA) with $\lambda_{excitation/emission}$ = 400/502 nm. A calibration curve was prepared using 5 to 100 μmol/L of CFA following the same procedure as above. Data fits the compartmental model using a PK Solver 2.0 program.

Supplemental Reference

1. Tsai SY, Chen SC. A fluorometric assay of trans-cinnamaldehyde in cinnamon. J Nat Prod. 1984; 47: 536-8.
